# Predictive modelling of transport decisions and resources optimisation in pre-hospital setting using machine learning techniques

**DOI:** 10.1371/journal.pone.0301472

**Published:** 2024-05-03

**Authors:** Hassan Farhat, Ahmed Makhlouf, Padarath Gangaram, Kawther El Aifa, Ian Howland, Fatma Babay Ep Rekik, Cyrine Abid, Mohamed Chaker Khenissi, Nicholas Castle, Loua Al-Shaikh, Moncef Khadhraoui, Imed Gargouri, James Laughton, Guillaume Alinier

**Affiliations:** 1 Ambulance Service, Hamad Medical Corporation, Doha, Qatar; 2 Faculty of Sciences, University of Sfax, Sfax, Tunisia; 3 Faculty of Medicine “Ibn El Jazzar”, University of Sousse, Sousse, Tunisia; 4 College of Engineering, Qatar University, Doha, Qatar; 5 Faculty of Health Sciences, Durban University of Technology, Durban, South Africa; 6 Laboratory of Screening Cellular and Molecular Process, Centre of Biotechnology of Sfax, University of Sfax, Sfax, Tunisia; 7 Higher Institute of Biotechnology, University of Sfax, Sfax, Tunisia; 8 Faculty of Medicine, University of Sfax, Sfax, Tunisia; 9 University of Hertfordshire, Hatfield, United Kingdom; 10 Weill Cornell Medicine-Qatar, Doha, Qatar; 11 Northumbria University, Newcastle upon Tyne, United Kingdom; UNITEN: Universiti Tenaga Nasional, MALAYSIA

## Abstract

**Background:**

The global evolution of pre-hospital care systems faces dynamic challenges, particularly in multinational settings. Machine learning (ML) techniques enable the exploration of deeply embedded data patterns for improved patient care and resource optimisation. This study’s objective was to accurately predict cases that necessitated transportation versus those that did not, using ML techniques, thereby facilitating efficient resource allocation.

**Methods:**

ML algorithms were utilised to predict patient transport decisions in a Middle Eastern national pre-hospital emergency medical care provider. A comprehensive dataset comprising 93,712 emergency calls from the 999-call centre was analysed using R programming language. Demographic and clinical variables were incorporated to enhance predictive accuracy. Random Forest (RF), Support Vector Machine (SVM), Extreme Gradient Boosting (XGBoost), and Adaptive Boosting (AdaBoost) algorithms were trained and validated.

**Results:**

All the trained algorithm models, particularly XGBoost (Accuracy = 83.1%), correctly predicted patients’ transportation decisions. Further, they indicated statistically significant patterns that could be leveraged for targeted resource deployment. Moreover, the specificity rates were high; 97.96% in RF and 95.39% in XGBoost, minimising the incidence of incorrectly identified “Transported” cases (False Positive).

**Conclusion:**

The study identified the transformative potential of ML algorithms in enhancing the quality of pre-hospital care in Qatar. The high predictive accuracy of the employed models suggested actionable avenues for day and time-specific resource planning and patient triaging, thereby having potential to contribute to pre-hospital quality, safety, and value improvement. These findings pave the way for more nuanced, data-driven quality improvement interventions with significant implications for future operational strategies.

## Introduction

Ambulance services, often called “Emergency Medical Services” (EMS), are undergoing significant transformations globally, reflecting contemporary healthcare systems’ complex, dynamic challenges. In a world where rapid technological advancements often outpace the ability of traditional healthcare infrastructure to adapt, the pressing imperatives include effective and efficient medical intervention strategies. EMS face multifactorial challenges, including increasing patient volumes, finite resources, and the escalating need for speed and precision in life-saving interventions [1]. These challenges are particularly pronounced in multinational societies like Qatar, where a diverse demographic landscape adds layers of complexity to emergency medical care.

However, the utility of advanced techniques, such as machine learning (ML), assumes immense significance. The application of ML, a subfield of artificial intelligence, serves as a catalyst for deep data exploration—unearthing patterns and correlations that often remain invisible in traditional analyses [2]. In an environment where every minute counts, the capacity to predict medical needs and allocate resources judiciously is a dynamic process. ML‘s predictive analytics provide an innovative approach to bridging existing gaps in the EMS domain, offering potentially transformative solutions to both old and new challenges [3].

In the literature, the integration of ML in EMS marked a significant shift towards more efficient and responsive healthcare systems. The role of predictive analytics in healthcare, and specifically in EMS. For instance, research demonstrated that ML helped enhance diagnostic processes. Their study showed that ML algorithms could significantly improve the accuracy and speed of medical diagnoses, particularly in complex cases like cancer detection. This advancement in diagnostics was crucial for early intervention and effective treatment planning. Further, other researchers highlighted how ML is used in patient care management by personalising patient care plans, leading to better patient outcomes and more efficient use of healthcare resources. This aspect of ML aligns with the overarching goal of improving healthcare delivery and patient satisfaction. It also helped predict and manage public health crises, such as pandemics. Their research illustrates how ML models can forecast disease spread patterns, aiding in the formulation of effective public health responses. It helped forecast medical emergencies and anticipate specific trends and emergencies, enabling proactive resource allocation. Recent studies have highlighted the potential of ML in transforming EMS operations by enhancing response times and resource allocation in EMS and reducing. Similarly, other studies indicated that ML algorithms could predict high-demand areas, allowing for better ambulance positioning and quicker response time, as well as resource optimisation. Other studies revealed that ML could lead to cost savings in healthcare by reducing unnecessary interventions aligning with the goal of value improvement, indicating that ML can contribute to cost reduction and quality enhancement in healthcare services.

Qatar’s multinational population makes it a uniquely challenging environment for the emergency care service [4]. Hamad Medical Corporation Ambulance Service (HMCAS) is the leading provider in this setting and continually evolves its healthcare infrastructure. This makes HMCAS a particularly relevant case study for integrating ML techniques into its operational system. The rich data generated in such a complex environment are fertile grounds for ML algorithms to tease out nuanced insights that can inform more effective resource allocation strategies. With healthcare costs soaring worldwide, there is a growing need for value improvement (VI), a quality improvement (QI) concept that aims not only at cost reduction but also at enhancing the quality of healthcare services [5]. ML fits neatly into this VI culture. ML could contribute to resource optimisation by facilitating advanced predictive analytics of patient transport decisions, ultimately leading to fiscal savings and improved healthcare outcomes. To our knowledge, no previous study approached the patients’ transport decisions in a pre-hospital setting using ML.

The study aimed to investigate the role of ML algorithms in predicting patients’ transport decisions, which would allow optimal resource allocation in HMCAS in Qatar.

## Method

### Setting and source of data

A retrospective quantitative analysis with predictive modelling was conducted on 93,712 pre-hospital emergency calls received by HMCAS between January 1st and May 31st, 2023. The electronic patient care record (ePCR) system, overseen by the HMCAS Business Intelligence (BI) division, served as the data source. Compliance with the Transparent Reporting of a multivariable prediction model for Individual Prognosis Or Diagnosis (TRIPOD) guidelines was maintained throughout the study for machine learning-based analyses [6]. Ethical approval was obtained from the Hamad Medical Corporation Medical Research Centre (Reference: MRC-01-22-264). R-Studio was employed for data processing and analysis.

### Participants

The criteria for inclusion encompassed all 999 emergency calls leading to the dispatch of an ambulance, during which a paramedic conducted an on-scene patient evaluation, irrespective of whether the patient was transported to a hospital or not. Exclusion criteria were the cases involving a deceased individual and when the 999 call was made by a healthcare facility.

### Statistical analysis

#### Predictors and outcome variable

The variable “Handover” was considered the outcome variable. It included a binary outcome, which is whether a patient, after receiving emergency treatment by paramedics on scene, was conveyed to a healthcare facility (“Transported” = 1) or not (“Not Transported” = 0).

The continuous predictors were “Age”, “Weight”, and the hour when the 999 call was received (“Hour_received”).

The categorical variables were nationalities categories (“Nationality_CAT”), the entity managing the 999 calls or “CFS_Owner” (A “Call For Service” can be attributed to the Police, Civil Defense, or HMCAS), Program Question Answer^TM^ (ProQA) “ProtocolName”, “Unit_Type”, “DispatchType”, which is determined according to the ProQA protocol outcome, “ProvisonalDiagnoses_CAT”, and co-morbidities such as “Seizure”, “Asthma”, Diabetes Mellitus “DM”, Chronic obstructive pulmonary disease (“COPD”), “CurrentlyPregnant”, Cerebral Vascular Accident (“CVA”), “Hypertension”, Cardio Artery Disease (“CAD”), “Other”, and Unknown.

#### Data pre-processing

Effective data analysis in a medical context hinges upon the quality and coherence of the underlying data [7]. Pre-processing steps were implemented to ensure the dataset’s reliability, interpretability, and clinical relevance by renaming, recoding, transforming variables, and handling missing values.

First, the variables’ renaming was conducted to make them understandable in a manageable format. This step is foundational, ensuring that subsequent operations are intuitive and less error-prone. This was achieved by mapping column names to more descriptive terms. This included the variables “ProtocolName”, “ProtocolCode”, “DispatchType”, “ProvisonalDiagnosis”, and “Seizure”. Second, existing values under the variables were recoded to a more understandable form, such as the values under “ProtocolName” that were recoded into [the protocol name and its ProQA™ code between “()”] as defined by the International Academy of Emergency Medical Dispatch [8]. Third, data transformation was performed. For example, values under the variables “ProtocolName” and “DispatchType” were altered based on the corresponding value in the “CFS_Owner” field. Data was restructured to represent the calendar week numbers.

Further, in the transformation, recategorisation of several variables such as “TransportedTo”, the hospital facility “PatientTriageArea”, “ProvisonalDiagnosis”, and “Nationality_CAT” was performed. Fourth, the variables not required after transformation were removed. Fifth, the missing values were assessed by generating a plot to allow the visualisation of the ‘missingness’ patterns across variables [9]. This was a twofold process: initially, to assess the extent and nature of missing data and second, to verify the effectiveness of the missing values management technique. Missing data were managed using the Multiple Imputation by Chained Equations (MICE) algorithm. MICE is a statistical technique that runs multiple simulations to fill in the missing data, offering a more accurate representation of the data landscape [9]. It uses all available variables to estimate the missing ones, proceeding variable by variable iteratively repeated until the dataset is complete. This process culminates in several datasets that are analysed collectively, enhancing the reliability of the research outcomes. “Location_LAT’ and “Location_Long” were excluded from the MICE process as they were only utilised to plot the patient transport/not transported decision map. For continuous variables, the MICE imputation method deployed was the Predictive Mean Matching (PMM), whereas the Classification and Regression Trees (CART) MICE method was utilised for the categorical variables [10]. The imputed values were integrated into the original dataset, fulfilling the replacement of missing values for comprehensive data.

Outliers were assessed by designing boxplots. They evinced no discernible outliers, thus obviating the need for further outlier removal steps. Hence, the initial data was deemed devoid of outliers and was retained for subsequent analysis. Feature selection was conducted by employing two automated methods to ensure the robustness of the chosen variables. The first method was the Random Forest (RF) algorithm. The second method was the Recursive Feature Elimination (RFE) using the “Wrapped Method” [11]. RFE is used to evaluate a combination of features and assign a score based on model accuracy considering the interactions between features [11]. The Wrapped Method helps improve the RFE’s performance by automatically selecting the most important features, thereby making the model more accurate and efficient [12].

Further, categories with low counts, such as the non-EMS ProQA™ call-taking protocols, such as “Maritime”, “Fire”, and “Rescue”, under the “ProtocolName” variable, were grouped together to avoid algorithmic errors associated with sparse data during ML analysis. Categorical variables were converted into numerical codes to avoid causing errors when building the models.

It is important to note that despite preliminary feature selection, a substantial correlation existed among variables, as confirmed by Chi-square and Wilcoxon rank-sum tests (S1 File). Variables selected for inclusion were not determined solely by statistical tests; they were also influenced by the researchers’ expertise and what was previously employed in the literature [13]. Other epidemiological analyses that identified significant associations with co-morbidities further informed the selection, including the table in S1 File. Therefore, in addition to “Handover,” which served as the outcome variable, the final list of variables retained for supervised predictive modelling included “Unit_Type,” “Region,” “ProtocolName,” “WeekNumber,” “Hour_Received,” “LocationType,” “WeekDay,” “Age,” “Gender,” “Nationality_CAT,” “ProvisonalDiagnoses_CAT,” “Hypertension,” and “DM.”

### Predictive modelling

Supervised predictive modelling (PM) was adopted for this study.

The pre-processed dataset was segregated into training (80%; n = 70,783.20) and test subsets (20%; n = 17,695.80). This partitioning is critical for unbiased model evaluation and preventing overfitting [14].

Parallel computing (PC) was utilised to speed up the data analysis process. A PC allows multiple calculations simultaneously by taking advantage of the multiple cores in a processor [15]. This approach benefits complex computations, helping us obtain results more quickly and efficiently.

Hyperparameter tuning was used to optimise the performance of the ML algorithms [16]. This enables the creation of the most effective model for predicting the transport decisions variable with the highest accuracy, increasing the reliability of the results.

Further, four algorithms were employed to train the data: 1) RF utilised a ten-fold cross-validation [14]. This approach randomly divided the training dataset into ten equal-sized subsets. The RF algorithm was trained on nine subsets and tested on the remaining one. This procedure was repeated ten times using a different subset for validation. 2) Support Vector Machine (SVM) was enhanced with a Radial Basis Function (RBF) Kernel. RBF Kernel optimises model performance by measuring the similarity between data points and examining their relative positions in a defined set of characteristics [17]. These characteristics are the pre-determined variables identified as relevant to the model’s predictive accuracy [17]. 3) The eXtreme Gradient Boosting (XGBoost) was adopted using its built cross-validation system, which provides a robust and highly accurate algorithmic approach [18]. 4) Adaptive Boosting (AdaBoost) was also utilised. AdaBoost allows boosting the performance of the models by iteratively correcting their errors, offering a straightforward yet effective ensemble method [18].

Each model was evaluated on several metrics such as Accuracy, Sensitivity, Specificity, Matthews Correlation Coefficient (MCC), and Area Under the Receiver Operating Curve (AUC). Prediction’s feature importance was assessed. Feature importance allows for identifying the most influential variables in the predictive models. The final predictive models were used to make predictions on the test data. Control charts, specifically p-charts and c-charts, were utilised for statistical quality control to monitor the performance of each model over time [19, 20]. Using the statistical process control (SPC) charts, the prediction models were summarised and visualised by day hour (“Hour_received”) and weekday to allow a better understanding of the predictive models’ performance at different times of the day and on different weekdays. This is crucial to assess the usefulness of these models in pre-hospital settings.

## Results

**Fig 1:**
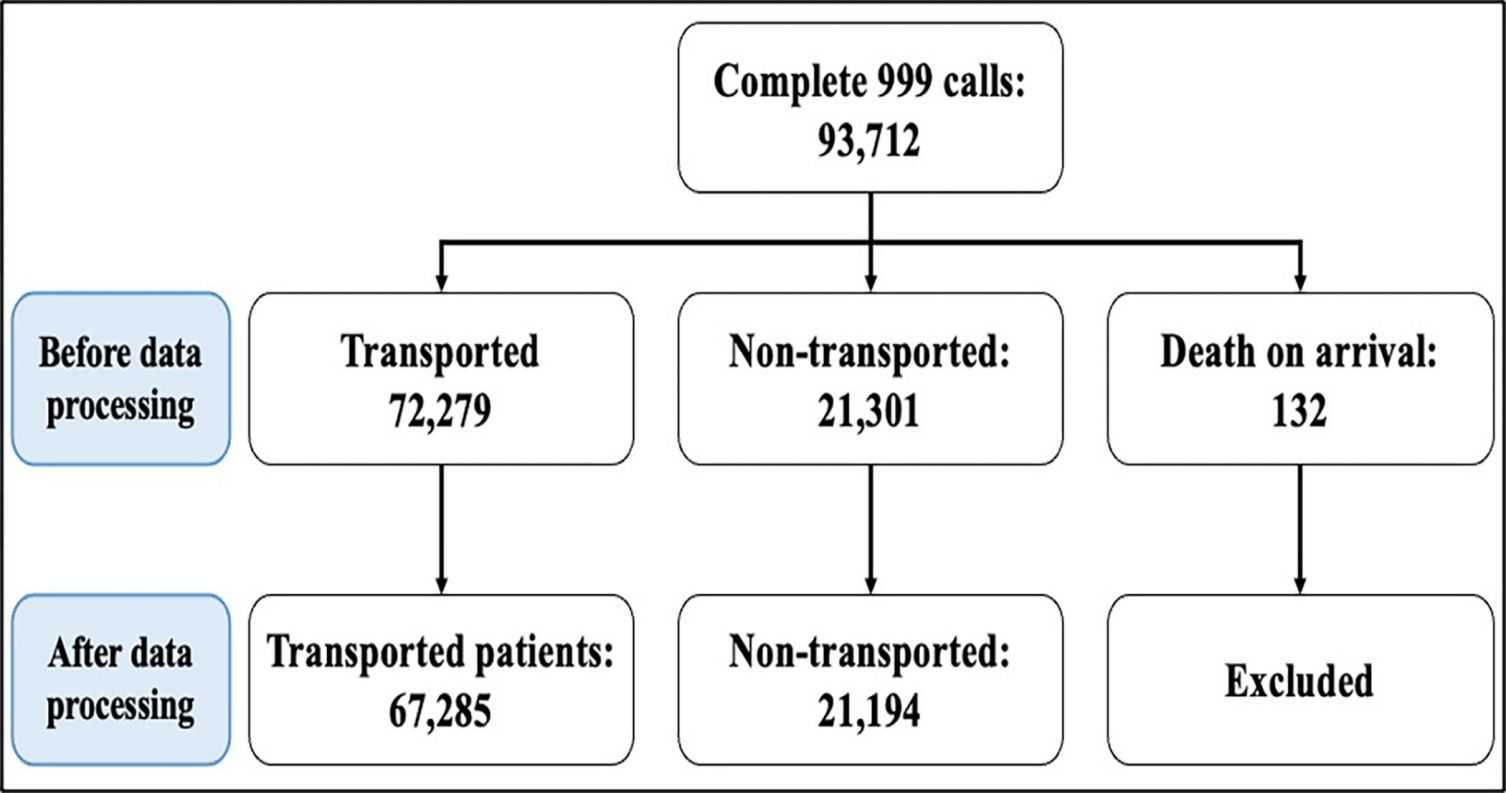
Geographical distribution of transported and not transported patients across Qatar. (This map was created by the first author using the “Leaflet” package in R. The data is available under the Open Database License).

From June to May 31st, 2023, 93,712 emergency calls were received, resulting in 72,279 patients being transported, 21,301 not being transported, and 132 cases identified as cases of death upon arrival on scene. After data pre-processing and removing the cases that did not comply with the inclusion criteria, 67,285 cases of transported patients and 21,194 related to patients who were not transported were retained for the predictive modelling (Fig 1).

“S2 File” presents the missing value percentages before and after the imputation.

“S3 File” presented boxplots generated to visualise the outliers within the continuous variables. No discernible outliers were visualised.

Automated feature selection methods were used. The Mean Decrease Accuracy (MDA) and Mean Decrease Gini (MDG) determined by RF and the scree-plot coefficients determined by RFE were plotted respectively in “S3 File”. For the RF method, the MDC indicates how much model accuracy decreases when a feature is randomly permuted. Higher values imply that the feature is important. The MDG measured how each feature contributed to the homogeneity of the nodes and leaves in the resulting Random Forest. The RFE method evaluated the effectiveness of different feature sets in predicting an outcome. Lower Root Mean Square Error and Mean Absolute Error values, along with R^2^ values closer to 1, signal better feature sets for accurate predictions; the smaller the associated standard deviations, the more consistently reliable the selected feature is. Although the RFE does not directly provide the variable names in its table and attributes them numbers instead, it provides the most significant variable at the end. The tables in “S4 File” indicated that features such as provisional diagnoses (“ProvisonalDiagnoses_CAT”), priority to hospital (“PriorityToHospital”), ProQA™ call-taking protocol name (“ProtocolName”), and patient triage area (“PatientTriageArea”) were predominant for accurate predictive modelling. Hence, the variables included in the modelling were selected based on the researchers’ clinical experience and operational relevance.

For the predictive modelling, “Table 1” evaluated the four ML algorithms utilised in this study to predict whether patients who call 999 will be transported to a hospital or not. Each of the models’ performance metrics (Accuracy, Sensitivity, Specificity, Kappa, and McNemar’s Test p-value) were determined. Accuracy denotes the proportion of true positives and true negatives among the total observations. The “No Information Rate (NIR) and p-value” indicate the baseline model’s accuracy, which in this case is 76.05%. The p-value suggests that the models are statistically significant in improving prediction compared to a no-information model. Sensitivity and Specificity represent the model’s ability to identify “true positives” and “true negatives” correctly. High Specificity across the models (97.69% in Random Forest to 95.39% in XGBoost) suggests fewer false positives. Kappa offers a chance-corrected measure of agreement between observed and predicted classifications. Higher Kappa suggests better performance. XGBoost had the best Kapp value (k = 0.46) and then RF (k = 0.42). McNemar’s Test helps us understand whether the ML model was genuinely helpful in making reliable predictions or if it was by chance. The p-value was <0.01 for all models, suggesting they are significantly better at making correct predictions. The MCC represents the quality of binary classifications. The closest to 100%, the better the prediction. XGBoost had the best MCC (48.47%) followed by RF (47.02%). For the AUC (85.05% for XGBoost and 84.87% for RF), the higher the value, the better the model’s performance. Positive Predictive Value, Negative Predictive Value, and Balanced Accuracy are ways to better understand how good a model is at making predictions. True Negatives refer to those cases when patients were “Not Transported” in reality and correctly predicted by the algorithm. False Positives refer to cases when patients were “Not Transported” but were incorrectly predicted as “Transported” by the algorithm. Lastly, False Negatives refer to “Transported” cases but were incorrectly predicted as “Not Transported”.

**Table 1:** Predictive models performance metrics.

Random_Forest	XGBoost	AdaBoost	Support_Vector_Machine
Accuracy: 82.90%	Accuracy: 83.10%	Accuracy: 78.25%	Accuracy: 76.65%
95% CI: (82.3%, 83.43%)	95% CI: (82.54%, 83.65%)	95% CI: (77.64%, 78.86%)	95% CI: (76.02%, 77.27%)
No Information Rate (NIR): 76.05%	No Information Rate (NIR): 76.05%	No Information Rate **(**NIR): 76.05%	No Information Rate (NIR): 76.05%
p-value [Acc* > NIR]: < 0.01	p-value [Acc^*^ > NIR]: < 2.2×10^−16^	p-value [Acc^*^ > NIR]: 2.033×10^−12^	p-value [Acc^*^ > NIR]: 0.032
Sensitivity: 35.87%	Sensitivity: 44.10%	Sensitivity: 17.39%	Sensitivity: 72.20%
Specificity: 97.69%	Specificity: 95.39%	Specificity: 97.42%	Specificity: 98.55%
Kappa: 0.42	Kappa: 0.46	Kappa: 0.20	Kappa: 0.08
Mcnemar’s Test p-value: < 0.01	Mcnemar’s Test p-value: < 0.01	Mcnemar’s Test p-value: < 0.01	Mcnemar’s Test p-value: < 0.01
Confusion Matrix and Statistics	Confusion Matrix and Statistics	Confusion Matrix and Statistics	Confusion Matrix and Statistics
Prediction 0 1	Prediction 0 1	Prediction 0 1	Prediction 0 1
0 1520 311	0 1869 621	0 737 347	0 306 200
1 2718 13146	1 2369 12836	1 3501 13110	1 3932 13257
Pos Pred Value: 83.01%	Pos Pred Value: 75.06%	Pos Pred Value: 67.99%	Pos Pred Value: 60.48%
Neg Pred Value: 82.87%	Neg Pred Value: 84.42%	Neg Pred Value: 78.93%	Neg Pred Value: 77.13%
Prevalence: 23.95%	Prevalence: 23.95%	Prevalence: 23.95%	Prevalence: 23.95%
Detection Rate: 8.59%	Detection Rate: 10.56%	Detection Rate: 4.17%	Detection Rate: 1.73%
Detection Prevalence: 10.35%	Detection Prevalence: 14.07%	Detection Prevalence: 6.13%	Detection Prevalence: 2.86%
Balanced Accuracy: 66.78%	Balanced Accuracy: 69.74%	Balanced Accuracy: 57.41%	Balanced Accuracy: 52.87%
‘Positive’ Class: 0	‘Positive’ Class: 0	“Positive” Class: 0	‘Positive’ Class: 0
MCC: 47.02%	MCC: 48.47%	MCC: 26.37%	MCC: 14.69%
AUC: 85.05%	AUC: 84.87%	AUC: NULL	AUC: NULL

**Fig 2:**
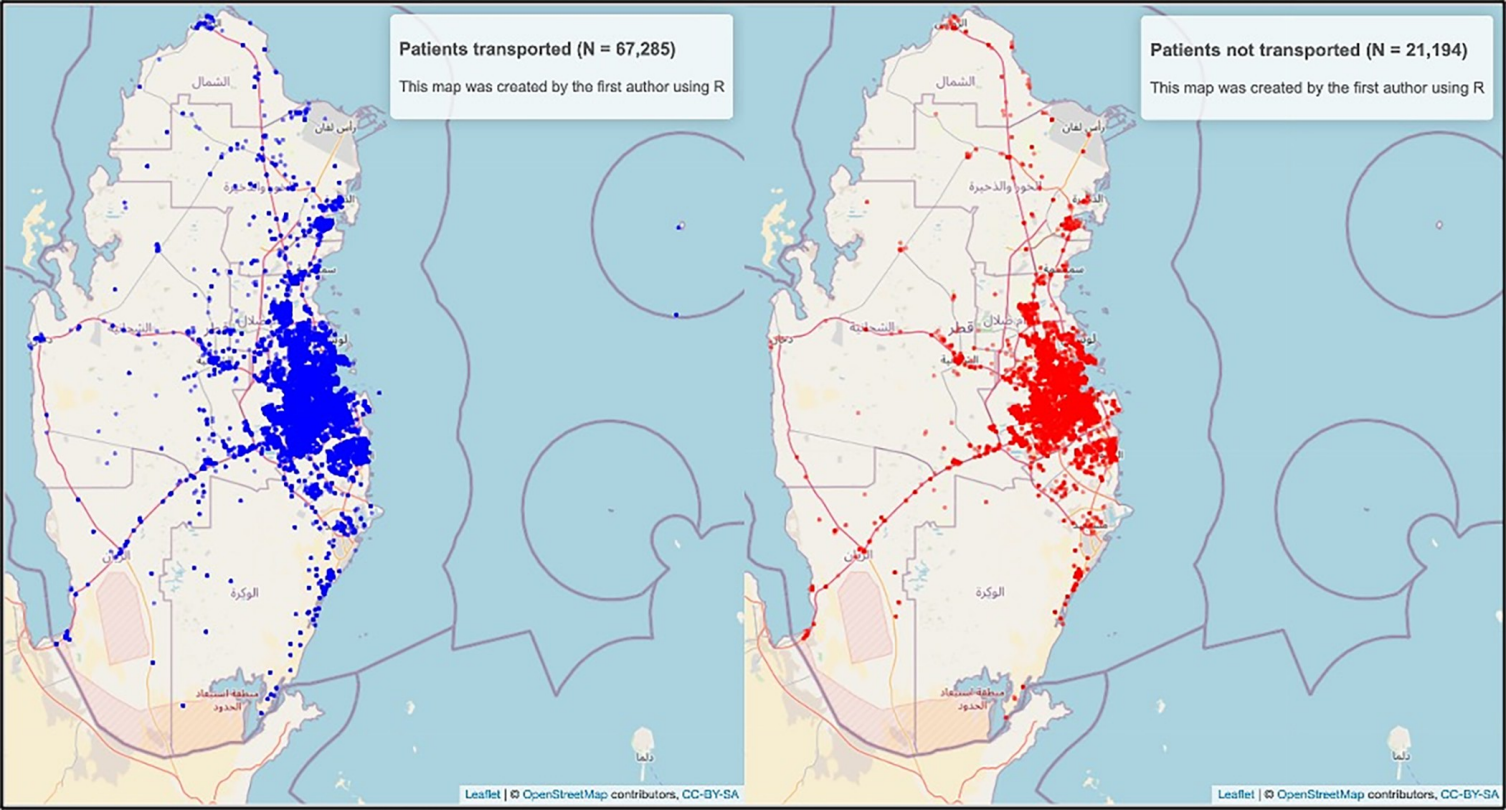
Confusion matrix plots of the four algorithms.

“Fig 2” shows a comparative evaluation of four ML algorithms was performed. RF demonstrated a robust sensitivity in identifying “Transported” cases with a high True Positive count (13,146). Still, it struggled to correctly predict the “Not Transported” cases since relatively low True Negative cases (n = 1,520) and high False Positive cases (n = 2,718) were predicted. XGBoost performed similarly, although with a slightly improved predicted True Negative rate (n = 1,869) but a considerable number of False Positives (n = 2,369) and False Negatives (n = 621). SVM, with an extremely high True Positive rate (1,3257), evidently failed in identifying “Not Transported” cases, giving an extremely low True Negative (n = 306) and high False Positive (n = 3,932) cases. AdaBoost showed a more balanced performance than SVM, with a commendable True Positive rate (n = 13,110) and a better True Negative rate (n = 737), although it also predicted a significant number of False Positives (n = 3,501) and False Negatives (n = 347).

**Fig 3:**
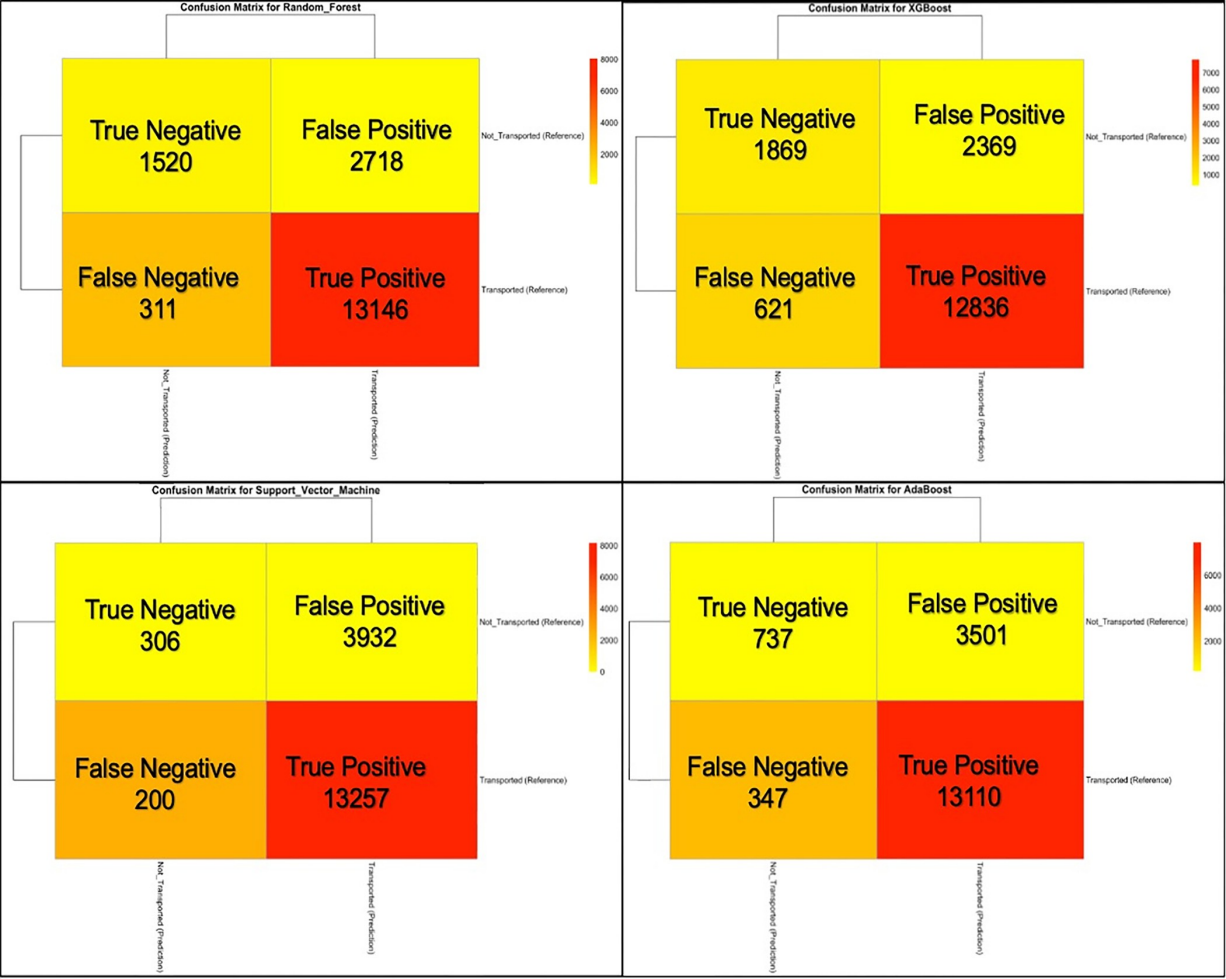
Feature importance of the four machine learning models.

Additionally, “Fig 3” was created to identify the most important variables in the predictive models. The call-tacking “ProtocolName” and “ProvisonalDiagnoses_CAT” were the most significant features across all variables.

**Fig 4:**
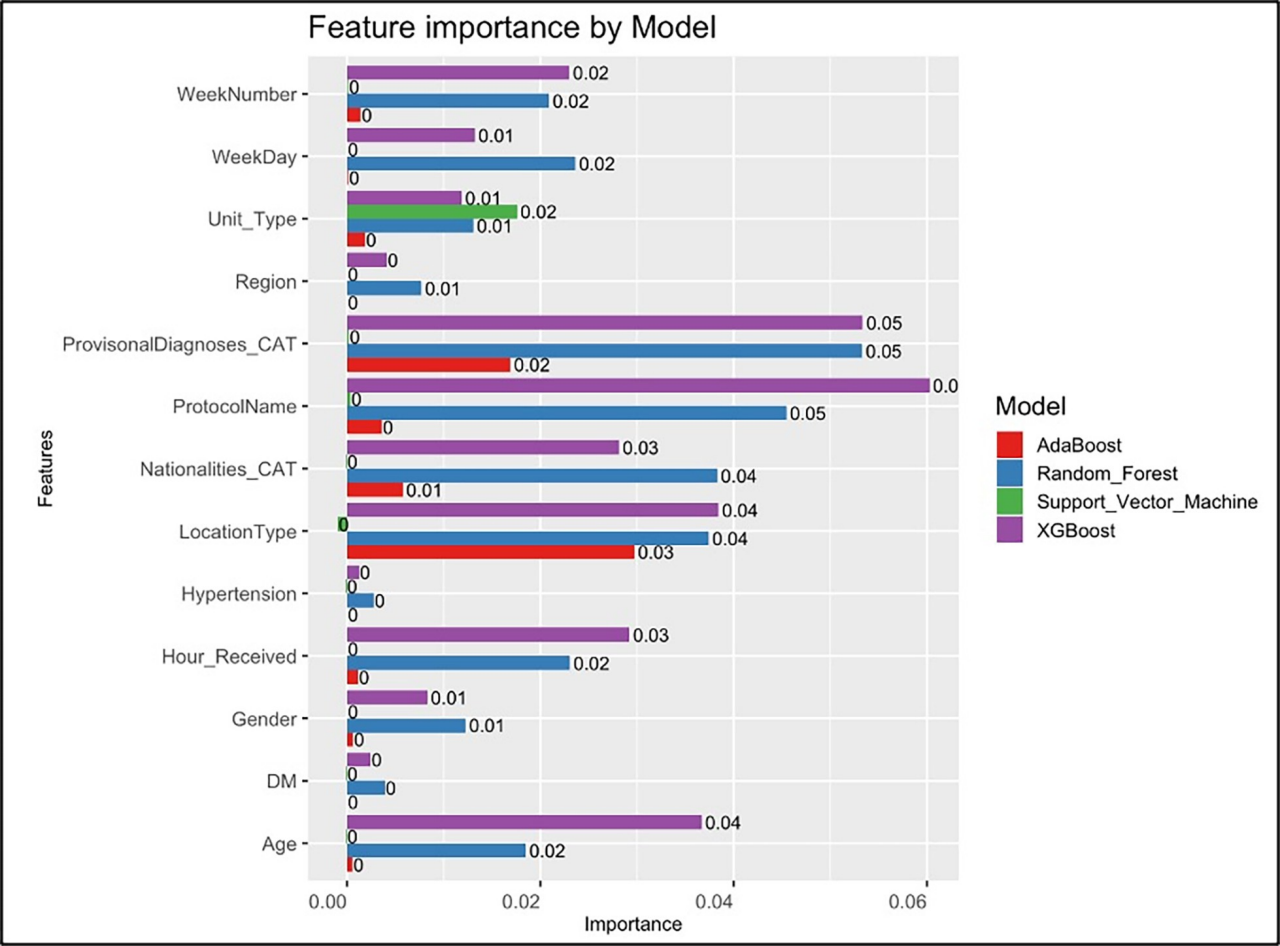
Statistical process control of the prediction data of the hourly patients not transported per model.

**Fig 5:**
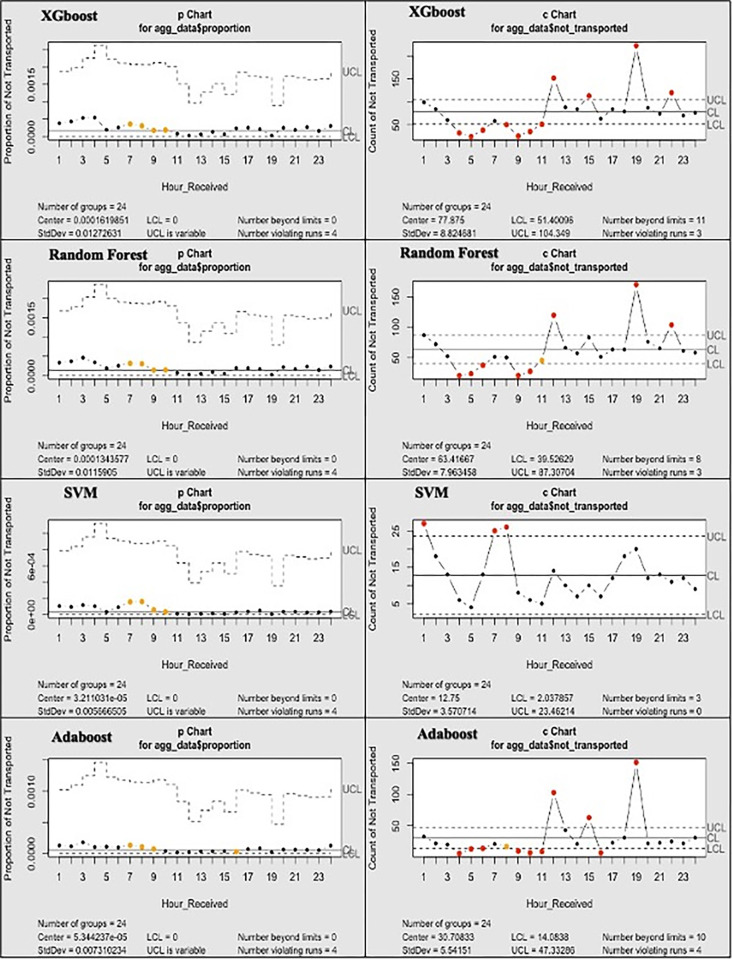
Hourly and weekly number of patients predicted to be transported to the hospital for all models.

In “Figs 4 and 5”, and “S5 File”, the prediction data showcases the frequency of 999 calls categorised into (Yes = Transported) and (No = Not Transported) across different days of the week and hours of the day. For RF and XGBoost, the best performing models, Sunday’s 999 call rates remained relatively stable between both models, with only a slight decrease in XGBoost, whilst certain days like Monday showed more variability. The late-night and early-morning hours consistently have fewer 999 calls across all days in both datasets. The frequency of transported patients tends to be higher during the day than at night for almost all days of the week (Except on Fridays). Peaks in the transported patients can be seen during late morning to early afternoon hours, notably for Mondays and Wednesdays. For the “Not Transported” patients, the frequency generally remains lower compared to “Transported” patients but still shows some hourly variations.

The following examples are illustrated to explain how to use the data in “S5 File” for the prediction of 999 calls using the XGBoost algorithm, also considering the other predictors in the model:

- **Monday at Hour 0**: On Mondays at midnight, we can expect approximately 50 are predicted to be transported, while 15 will likely not be transported.
- **Wednesday at Hour 11**: On Wednesdays at 11 AM, we can expect approximately 385 to be transported, while 61 will likely not be transported.
- **Friday at Hour 18**: On Fridays at 6 PM, we can expect approximately about 50 are predicted to be transported, while 12 will likely not be transported.
- **Sunday at Hour 22**: On Sundays at 10 PM, we can expect approximately 62 are predicted to be transported, while 14 will likely not be transported.

## Discussion

In the feature selection process, variables such as “PatientTriageArea” and “PriorityToHospital” emerged as significant predictors for constructing a robust model. However, these variables were not incorporated into the final models for several reasons. Firstly, the area where a patient is triaged and the transport priority are frequently subject to change. Specifically, the patient triage area is often not determined until the ambulance is near the hospital. This is because patients’ medical conditions are dynamic, evolving during the transport journey, thereby affecting the final decision regarding identifying their destination area and the priority level, when the patient has already approved to be transported. Secondly, alternative feature selection methods, such as the Chi-square test in “S1 File”, revealed that most variables correlated highly with the outcome variable. This suggests that they, too, could substantially contribute to constructing a predictive model. Therefore, while automated feature selection techniques provide valuable insights, it is crucial also to consider other statistical and non-statistical measures to ensure a comprehensive understanding of how different variables could influence the model’s performance. This multifaceted approach enriches the robustness of the model, accommodating wider clinically and operationally significant factors like the call-taking protocol name. Moreover, prior studies have emphasised that feature selection serves as a guide towards pertinent variables, and features can be selected based on both their mutual information and correlation coefficients [11, 21, 22]. Nevertheless, the expertise of the researchers remains integral in selecting variables for inclusion in the predictive model. Ultimately, the model’s performance metrics serve as the definitive evaluation of its efficacy and reliability. While feature selection algorithms can inform variable importance, the researchers’ nuanced understanding and domain knowledge are indispensable for robust and clinically relevant modelling.

Effective decision-making is often encumbered by the lack of robust, predictive data analytics in pre-hospital care [23]. Integrating ML techniques into healthcare has foreshowed a new era of efficiency, predictability, and QI, particularly in pre-hospital settings [3, 24]. This study demonstrated the robustness of ML algorithms with high accuracy rates in predicting whether an emergency call would result in the patient being “Transported”’ or not. These findings have multiple implications for HMCAS, offering avenues for systemic QI interventions that were previously untapped.

Firstly, the predictive prowess of these algorithms can serve as a valuable tool for resource optimisation. When medical resources are finite, but demands are often unpredictable and urgent, allocating resources judiciously might be essential. In our case, RF and XGBoost models can help identify high-risk time periods likely to experience peaks in emergencies. By doing so, HMCAS can pre-position ambulances, personnel, and all other necessary equipment, thereby reducing response times and potentially increasing the effectiveness of the care provided. Many recent studies explored the potential for predictive analytics to improve pre-hospital medical teams’ performance [3, 25].

Secondly, the high specificity rates of these models can drastically reduce the incidence of false positives, ensuring that resources are allocated only where truly needed. In the context of patient transport decisions, false positives are erroneously predicting that a patient needs to be transported when they do not, which can lead to unnecessary use of pre-hospital resources. This strains healthcare systems and could divert critical resources from patients requiring urgent care. Therefore, even models with high specificity must be cautiously interpreted and continually validated to minimise these risks [26, 27].

The feature importance abilities of ML models (Fig 3) enabled the precise identification of key predictive variables, such as the ProQA™ protocol name and provisional diagnoses in our case. Based on these specific variables, this enhances the pre-hospital system’s proficiency in identifying patients at risk of not being transported. Such targeted predictive analysis can substantially ease financial and logistical pressures on pre-hospital care systems, for instance, by fine-tuning dispatch protocols to recognise better patients with low-acuity complaints who may request not to be transported. In such instances, alternative resources like “non-transport units” equipped with less costly medical equipment could be allocated rather than dispatching emergency response units, which are over-equipped and crewed by highly qualified staff like critical care paramedics as they are designed for more critical patients [28, 29]. This approach may not only lead to fuel, workforce, and vehicle maintenance savings. It could also mitigate the risks associated with high-priority driving, with increased risk of road traffic accidents and under-triage associated with not transporting a patient [30–33]. It is crucial to highlight that numerous recent studies have accentuated the significant risks of failing to transport patients who have called for pre-hospital emergency assistance, thereby depriving them of advanced in-hospital care [34, 35].

Thirdly, ML algorithms are fundamentally adaptive and can continually evolve when updated with new data, offering dynamic models that can adjust to societal behaviour, technological progress or healthcare guidelines [36, 37]. This adaptability is crucial for continuous QI as it allows pre-hospital EMS to be agile and responsive to new challenges or shifts in pre-hospital care needs [38]. For example, the ML models could be updated to consider the emergence of new health crises such as pandemics, seasonal trends in certain medical conditions, or changes in urban layouts that can affect transport times. Incorporating these findings into operational protocols can potentially revolutionise the pre-hospital care paradigm. For instance, ambulances and medical teams could be pre-positioned strategically during identified peak hours, thus decreasing response times and enhancing patient outcomes [39, 40]. Additionally, staff rosters could be adapted to align with these temporal patterns, ensuring sufficient medical personnel are available during high-demand periods. The availability of advanced telemedicine options could also serve as alternative care pathways for non-transport cases, thus averting unnecessary pressure on hospitals.

Finally, it is important to note that while ML holds great promise for enhancing EMS capabilities, its effective implementation necessitates a multi-disciplinary approach that includes healthcare professionals, data scientists, and policymakers. It is not only about algorithmic sophistication but also about ethical considerations, data privacy, and regulatory compliance. Further, published studies cautioned against over-reliance on algorithms, highlighting ethical concerns such as data misuse, patient confidentiality, and the potential for algorithmic bias [41–43]. The incorporation of ML into pre-hospital EMS practices represents more than just a technological advance; it signifies a paradigm shift with the potential to fundamentally transform the benchmarks of emergency pre-hospital care [44, 45]. Considering these complexities, integrating continuous QI principles with ML becomes instrumental when implementing predictive pre-hospital care models. These QI methodologies, such as those derived from Deming and Kaizen principles, are helpful since they focus on iterative testing and constant monitoring, ensuring that the ML algorithms (**S6 File**), when deployed, remain accurate, ethical, and aligned with healthcare objectives, enabling real-time assessment and their fine-tuning [46, 47]. Concurrently, the culture of “continuous improvement” can perfectly merge with the adaptive nature of ML, encouraging a culture of regular feedback and instantaneous adjustments, creating a self-updating system that can mitigate risks and dynamically evolve to meet the ever-changing demands on pre-hospital care.

## Limitations

While the models demonstrated commendable accuracy rates, it is noteworthy to mention that their sensitivity rates, particularly in the case of SVM, dipped to an inadequate 7.2% highlighting existing limitations in identifying “true positives”. This suggests that the models are potentially more inclined towards avoiding false positives at the expense of missing out on genuine emergency cases [48]. In this study, while all four algorithms excelled in sensitivity by correctly predicting a high number of “Transported” cases, they showed varying degrees of specificity and error control, highlighting the need for further fine-tuning to improve overall performance. Such a gap in model performance highlights the pressing need for future research to refine these algorithms to achieve a more balanced interplay between sensitivity and specificity with new data. Moreover, missing values in the dataset could contribute to this issue. Filling in these gaps is more than a data-cleaning operation, it is also a fundamental step towards improving the robustness of predictive modelling. Missing data, if not addressed, could introduce bias and compromise the validity of the findings, thus mitigating the utility of ML in this critical context. Given this, we recommend that HMCAS reviews policies and procedures, specifically to address non-systematic missing data entry, which will pave the way for more robust data analysis and predictive modelling in the future.

## Conclusion

In conclusion, this study serves as a seminal exploration into utilising ML algorithms for predicting transport decisions, optimising resource allocation and improving the quality of pre-hospital emergency care. It presented compelling evidence for the efficacy of predictive modelling in differentiating “Transported” from “Not Transported” patients, thereby aiding in identifying their transport decision-making process in advance. While the study aligns with previous research in underlining the criticality of timely interventions and the need for nuanced, data-driven approaches, it also accentuates areas needing further refinement—namely, the improvement of model sensitivity and the imperative of handling missing data for more robust predictions. In doing so, the study sets the stage for future investigations that could refine these predictive algorithms and further enhance HMCAS’ responsiveness and effectiveness. The findings offer valuable contributions to personalised, efficient, and pre-hospital life-saving medical interventions as we move towards an increasingly data-centric healthcare paradigm.

## Supporting information

S1 File Summary statistics and test results of the variables for patients transported and not transported. (PDF)

S2 File Summary of the missing values before and after the imputation process. (PDF)

S3 File Boxplots for continuous variables data visualisation. (PDF)

S4 File Tables of the automated feature selection methods. (PDF)

S5 File Table of the four models’ predictions (Yes = Transported; No = Not Transported). (PDF)

S6 File The R-code that was utilised for the predictive modelling. (PDF)
