# Supplementary material for: Predictive modelling of transport decisions and resources optimisation in pre-hospital setting using machine learning techniques
Source: PLoS One. 2024 May 3;19(5):e0301472. doi: 10.1371/journal.pone.0301472 (PMC11068197; doi:10.1371/journal.pone.0301472)
Supplement: S1 File — (PDF) [file pone.0301472.s001.pdf]

1

**Characteristic**	**Not Transported**, N = 21,194	**95% CI**	**Transported**, N = 67,285	**95% CI**	**p-value**
**CFS_Owner**					**<0.001**
EMS	10,096 (48%)	47%, 48%	34,499 (51%)	51%, 52%	
No call taking/Not Recorded	8,084 (38%)	37%, 39%	23,193 (34%)	34%, 35%	
Other	3,014 (14%)	14%, 15%	9,593 (14%)	14%, 15%	
**ProtocolName**					**<0.001**
Abdominal pain (P1)	560 (2.6%)	2.4%, 2.9%	2,001 (3.0%)	2.8%, 3.1%	
Abnormal Behaviour (P25)	199 (0.9%)	0.82%, 1.1%	637 (0.9%)	0.88%, 1.0%	
Allergies_Envenomations (P2)	124 (0.6%)	0.49%, 0.70%	533 (0.8%)	0.73%, 0.86%	
Animal bites (P3)	0 (0%)	0.00%, 0.02%	54 (<0.1%)	0.06%, 0.11%	
Assault (P4)	426 (2.0%)	1.8%, 2.2%	728 (1.1%)	1.0%, 1.2%	
Back Pain (P5)	309 (1.5%)	1.3%, 1.6%	1,375 (2.0%)	1.9%, 2.2%	
Breathing problems (P6)	1,632 (7.7%)	7.3%, 8.1%	5,202 (7.7%)	7.5%, 7.9%	
Burns (P7)	84 (0.4%)	0.32%, 0.49%	337 (0.5%)	0.45%, 0.56%	
Cardiac arrest (P9)	33 (0.2%)	0.11%, 0.22%	517 (0.8%)	0.70%, 0.84%	
Chest pain (P10)	934 (4.4%)	4.1%, 4.7%	4,024 (6.0%)	5.8%, 6.2%	
Choking (P11)	75 (0.4%)	0.28%, 0.45%	124 (0.2%)	0.15%, 0.22%	
CO/CBRN (P8)	47 (0.2%)	0.16%, 0.30%	63 (<0.1%)	0.07%, 0.12%	
Convulsions (P12)	242 (1.1%)	1.0%, 1.3%	1,102 (1.6%)	1.5%, 1.7%	
Criminal Incidents (None_EMS)	40 (0.2%)	0.14%, 0.26%	67 (<0.1%)	0.08%, 0.13%	
CVA_TIA (P28)	49 (0.2%)	0.17%, 0.31%	248 (0.4%)	0.32%, 0.42%	
Diabetic Problems (P13)	274 (1.3%)	1.1%, 1.5%	366 (0.5%)	0.49%, 0.60%	
Drowning related (P14)	0 (0%)	0.00%, 0.02%	7 (<0.1%)	0.00%, 0.02%	
Electrocution (P15)	14 (<0.1%)	0.04%, 0.11%	20 (<0.1%)	0.02%, 0.05%	
Entrapments (P22)	91 (0.4%)	0.35%, 0.53%	384 (0.6%)	0.52%, 0.63%	
Eye problems (P16)	47 (0.2%)	0.16%, 0.30%	124 (0.2%)	0.15%, 0.22%	
Falls (P17)	351 (1.7%)	1.5%, 1.8%	2,478 (3.7%)	3.5%, 3.8%	
Fire (None_EMS)	10 (<0.1%)	0.02%, 0.09%	9 (<0.1%)	0.01%, 0.03%	
Headache (P18)	338 (1.6%)	1.4%, 1.8%	711 (1.1%)	0.98%, 1.1%	
Heart Problems (P19)	231 (1.1%)	0.96%, 1.2%	603 (0.9%)	0.83%, 0.97%	
Hemorrhage (P21)	143 (0.7%)	0.57%, 0.80%	783 (1.2%)	1.1%, 1.2%	
Maritime (None_EMS)	0 (0%)	0.00%, 0.02%	1 (<0.1%)	0.00%, 0.01%	
Miscarriage_Pregnancy (P24)	41 (0.2%)	0.14%, 0.27%	1,521 (2.3%)	2.2%, 2.4%	
No call taking/Not Recorded	8,084 (38%)	37%, 39%	23,193 (34%)	34%, 35%	
Others	31 (0.1%)	0.10%, 0.21%	463 (0.7%)	0.63%, 0.75%	
Penetrating Trauma (P27)	11 (<0.1%)	0.03%, 0.10%	77 (0.1%)	0.09%, 0.14%	
Poisoning (ingestion) (P23)	44 (0.2%)	0.15%, 0.28%	144 (0.2%)	0.18%, 0.25%	
Rescue (None_EMS)	0 (0%)	0.00%, 0.02%	6 (<0.1%)	0.00%, 0.02%	
RTA (P29)	2,513 (12%)	11%, 12%	8,479 (13%)	12%, 13%	
Sick person (P26)	2,818 (13%)	13%, 14%	5,529 (8.2%)	8.0%, 8.4%	
Traumatic Injuries (P30)	394 (1.9%)	1.7%, 2.1%	1,929 (2.9%)	2.7%, 3.0%	
Unconscious (P31)	947 (4.5%)	4.2%, 4.8%	3,257 (4.8%)	4.7%, 5.0%	
Unknown Problem (P32)	58 (0.3%)	0.21%, 0.36%	189 (0.3%)	0.24%, 0.32%	
**DispatchType**					**<0.001**
No call taking/Not Recorded	8,084 (38%)	37%, 39%	23,193 (34%)	34%, 35%	
Not in use	1 (<0.1%)	0.00%, 0.03%	0 (0%)	0.00%, 0.01%	
T	17 (<0.1%)	0.05%, 0.13%	23 (<0.1%)	0.02%, 0.05%	
UncompletedProQA	26 (0.1%)	0.08%, 0.18%	85 (0.1%)	0.10%, 0.16%	
X	4,170 (20%)	19%, 20%	8,337 (12%)	12%, 13%	
Y	6,285 (30%)	29%, 30%	24,338 (36%)	36%, 37%	
Z	2,611 (12%)	12%, 13%	11,309 (17%)	17%, 17%	

**PriorityToScene**					**<0.001**
P1	17,665 (83%)	83%, 84%	56,849 (84%)	84%, 85%	
P2	3,529 (17%)	16%, 17%	10,436 (16%)	15%, 16%	
**PriorityToHospital**					**<0.001**
Not Applicable	21,194 (100%)	100%, 100%	480 (0.7%)	0.65%, 0.78%	
P1	0 (0%)	0.00%, 0.02%	3,415 (5.1%)	4.9%, 5.2%	
P2	0 (0%)	0.00%, 0.02%	62,945 (94%)	93%, 94%	
P3	0 (0%)	0.00%, 0.02%	445 (0.7%)	0.60%, 0.73%	
**WeekNumber**					**<0.001**
W1	2,089 (9.9%)	9.5%, 10%	6,397 (9.5%)	9.3%, 9.7%	
W10	1,183 (5.6%)	5.3%, 5.9%	3,503 (5.2%)	5.0%, 5.4%	
W11	1,218 (5.7%)	5.4%, 6.1%	3,490 (5.2%)	5.0%, 5.4%	
W12	1,144 (5.4%)	5.1%, 5.7%	3,254 (4.8%)	4.7%, 5.0%	
W13	1,098 (5.2%)	4.9%, 5.5%	3,119 (4.6%)	4.5%, 4.8%	
W14	358 (1.7%)	1.5%, 1.9%	1,028 (1.5%)	1.4%, 1.6%	
W2	1,760 (8.3%)	7.9%, 8.7%	6,431 (9.6%)	9.3%, 9.8%	
W3	1,782 (8.4%)	8.0%, 8.8%	6,780 (10%)	9.9%, 10%	
W4	2,399 (11%)	11%, 12%	7,075 (11%)	10%, 11%	
W5	2,117 (10.0%)	9.6%, 10%	6,592 (9.8%)	9.6%, 10%	
W6	1,910 (9.0%)	8.6%, 9.4%	6,111 (9.1%)	8.9%, 9.3%	
W7	1,812 (8.5%)	8.2%, 8.9%	6,624 (9.8%)	9.6%, 10%	
W8	1,171 (5.5%)	5.2%, 5.8%	3,318 (4.9%)	4.8%, 5.1%	
W9	1,153 (5.4%)	5.1%, 5.8%	3,563 (5.3%)	5.1%, 5.5%	
**WeekDay**					**<0.001**
Friday	1,793 (8.5%)	8.1%, 8.8%	5,613 (8.3%)	8.1%, 8.6%	
Monday	3,207 (15%)	15%, 16%	11,172 (17%)	16%, 17%	
Saturday	4,620 (22%)	21%, 22%	14,339 (21%)	21%, 22%	
Sunday	2,150 (10%)	9.7%, 11%	7,030 (10%)	10%, 11%	
Thursday	3,197 (15%)	15%, 16%	9,208 (14%)	13%, 14%	
Tuesday	2,217 (10%)	10%, 11%	8,203 (12%)	12%, 12%	
Wednesday	4,010 (19%)	18%, 19%	11,720 (17%)	17%, 18%	
**Region**					**<0.001**
Rural	6,069 (29%)	28%, 29%	20,351 (30%)	30%, 31%	
Unknown	3 (<0.1%)	0.00%, 0.05%	34 (<0.1%)	0.04%, 0.07%	
Urban	15,122 (71%)	71%, 72%	46,900 (70%)	69%, 70%	
**LocationType**					**<0.001**
Airport	3,326 (16%)	15%, 16%	2,509 (3.7%)	3.6%, 3.9%	
Beach/Sea/Ocean	95 (0.4%)	0.36%, 0.55%	305 (0.5%)	0.40%, 0.51%	
Farm	37 (0.2%)	0.12%, 0.24%	236 (0.4%)	0.31%, 0.40%	
Home	10,526 (50%)	49%, 50%	38,571 (57%)	57%, 58%	
Industrial Area	125 (0.6%)	0.49%, 0.70%	1,632 (2.4%)	2.3%, 2.5%	
Other	1,048 (4.9%)	4.7%, 5.2%	2,256 (3.4%)	3.2%, 3.5%	
Public Area	760 (3.6%)	3.3%, 3.8%	2,193 (3.3%)	3.1%, 3.4%	
Recreation (Sport)	55 (0.3%)	0.20%, 0.34%	254 (0.4%)	0.33%, 0.43%	
School	360 (1.7%)	1.5%, 1.9%	1,248 (1.9%)	1.8%, 2.0%	
Street (Road)	4,319 (20%)	20%, 21%	14,048 (21%)	21%, 21%	
Work	543 (2.6%)	2.4%, 2.8%	4,033 (6.0%)	5.8%, 6.2%	
**Unit_Type**					**<0.001**
Alpha	15,204 (72%)	71%, 72%	46,508 (69%)	69%, 69%	
Bravo	1,474 (7.0%)	6.6%, 7.3%	1,010 (1.5%)	1.4%, 1.6%	
Charlie	1,354 (6.4%)	6.1%, 6.7%	6,534 (9.7%)	9.5%, 9.9%	
Delta	1,421 (6.7%)	6.4%, 7.1%	4,907 (7.3%)	7.1%, 7.5%	
LF	95 (0.4%)	0.36%, 0.55%	646 (1.0%)	0.89%, 1.0%	

Other	1,646 (7.8%)	7.4%, 8.1%	7,680 (11%)	11%, 12%	
**Gender**					<0.001
Female	8,505 (40%)	39%, 41%	23,210 (34%)	34%, 35%	
Male	12,689 (60%)	59%, 61%	44,075 (66%)	65%, 66%	
**Nationalities_CAT**					**<0.001**
East Asia & Pacific	968 (4.6%)	4.3%, 4.9%	3,699 (5.5%)	5.3%, 5.7%	
Europe & Central Asia	936 (4.4%)	4.1%, 4.7%	1,565 (2.3%)	2.2%, 2.4%	
GCC Other	893 (4.2%)	3.9%, 4.5%	2,513 (3.7%)	3.6%, 3.9%	
Latin America & Caribbean	45 (0.2%)	0.16%, 0.29%	90 (0.1%)	0.11%, 0.17%	
MENA	5,363 (25%)	25%, 26%	14,463 (21%)	21%, 22%	
North America	139 (0.7%)	0.55%, 0.78%	244 (0.4%)	0.32%, 0.41%	
Other	805 (3.8%)	3.5%, 4.1%	2,669 (4.0%)	3.8%, 4.1%	
Qatar	5,094 (24%)	23%, 25%	11,187 (17%)	16%, 17%	
South Asia	5,052 (24%)	23%, 24%	24,007 (36%)	35%, 36%	
Sub-Saharan Africa	1,899 (9.0%)	8.6%, 9.4%	6,848 (10%)	10%, 10%	
**Age**	33 (24, 45)	35, 36	34 (25, 45)	36, 36	**<0.001**
**Weight**	70 (61, 80)	70, 70	75 (65, 80)	71, 72	**<0.001**
**ProvisonalDiagnoses_CAT**					**<0.001**
Acute trauma	908 (4.3%)	4.0%, 4.6%	5,714 (8.5%)	8.3%, 8.7%	
Allergy/Anaphylaxis	131 (0.6%)	0.52%, 0.74%	792 (1.2%)	1.1%, 1.3%	
Asthma	313 (1.5%)	1.3%, 1.7%	949 (1.4%)	1.3%, 1.5%	
Burn	118 (0.6%)	0.46%, 0.67%	507 (0.8%)	0.69%, 0.82%	
Cardiac Arrest	0 (0%)	0.00%, 0.02%	751 (1.1%)	1.0%, 1.2%	
Cardiovas_ACS	142 (0.7%)	0.57%, 0.79%	1,802 (2.7%)	2.6%, 2.8%	
Cardiovas_Other	0 (0%)	0.00%, 0.02%	2 (<0.1%)	0.00%, 0.01%	
Cardiovas_SupVent	27 (0.1%)	0.09%, 0.19%	408 (0.6%)	0.55%, 0.67%	
Cardiovas_Vent	1 (<0.1%)	0.00%, 0.03%	18 (<0.1%)	0.02%, 0.04%	
Chronic Condition	342 (1.6%)	1.5%, 1.8%	1,941 (2.9%)	2.8%, 3.0%	
COPD	9 (<0.1%)	0.02%, 0.08%	75 (0.1%)	0.09%, 0.14%	
COVID19_Related	108 (0.5%)	0.42%, 0.62%	584 (0.9%)	0.80%, 0.94%	
Croup/Epiglottitis	3 (<0.1%)	0.00%, 0.05%	117 (0.2%)	0.14%, 0.21%	
CVA/TIA	14 (<0.1%)	0.04%, 0.11%	433 (0.6%)	0.59%, 0.71%	
DOA	1 (<0.1%)	0.00%, 0.03%	206 (0.3%)	0.27%, 0.35%	
Electrocution	3 (<0.1%)	0.00%, 0.05%	14 (<0.1%)	0.01%, 0.04%	
Endocrinology_Other	0 (0%)	0.00%, 0.02%	6 (<0.1%)	0.00%, 0.02%	
Envenomation	5 (<0.1%)	0.01%, 0.06%	30 (<0.1%)	0.03%, 0.06%	
FBAO	14 (<0.1%)	0.04%, 0.11%	65 (<0.1%)	0.08%, 0.12%	
Fever	1,655 (7.8%)	7.5%, 8.2%	4,911 (7.3%)	7.1%, 7.5%	
GI	966 (4.6%)	4.3%, 4.8%	4,610 (6.9%)	6.7%, 7.0%	
GU	27 (0.1%)	0.09%, 0.19%	456 (0.7%)	0.62%, 0.74%	
Heat_related	6 (<0.1%)	0.01%, 0.06%	52 (<0.1%)	0.06%, 0.10%	
Hemothorax	0 (0%)	0.00%, 0.02%	13 (<0.1%)	0.01%, 0.03%	
Hyperglycemia	139 (0.7%)	0.55%, 0.78%	487 (0.7%)	0.66%, 0.79%	
Hypertension	53 (0.3%)	0.19%, 0.33%	550 (0.8%)	0.75%, 0.89%	
Hypoglycemia	227 (1.1%)	0.94%, 1.2%	215 (0.3%)	0.28%, 0.37%	
Low Acuity Problem_Medical	10,568 (50%)	49%, 51%	19,123 (28%)	28%, 29%	
Low Acuity Problem_Trauma	2,655 (13%)	12%, 13%	9,596 (14%)	14%, 15%	
Near drowining	1 (<0.1%)	0.00%, 0.03%	2 (<0.1%)	0.00%, 0.01%	
Neurology_Other	1,140 (5.4%)	5.1%, 5.7%	2,300 (3.4%)	3.3%, 3.6%	
OBS_GYN	32 (0.2%)	0.11%, 0.22%	2,222 (3.3%)	3.2%, 3.4%	
Other	282 (1.3%)	1.2%, 1.5%	2,718 (4.0%)	3.9%, 4.2%	
Pneumothorax	0 (0%)	0.00%, 0.02%	5 (<0.1%)	0.00%, 0.02%	
Respiratory_Infection	569 (2.7%)	2.5%, 2.9%	2,287 (3.4%)	3.3%, 3.5%	

Respiratory_Other	308 (1.5%)	1.3%, 1.6%	506 (0.8%)	0.69%, 0.82%	
Seizure	104 (0.5%)	0.40%, 0.60%	1,424 (2.1%)	2.0%, 2.2%	
Shock	0 (0%)	0.00%, 0.02%	66 (<0.1%)	0.08%, 0.13%	
Syncope	174 (0.8%)	0.71%, 0.95%	598 (0.9%)	0.82%, 0.96%	
Toxicology	126 (0.6%)	0.50%, 0.71%	535 (0.8%)	0.73%, 0.87%	
Transport	23 (0.1%)	0.07%, 0.17%	195 (0.3%)	0.25%, 0.33%	
**TransportedTo**					**<0.001**
Airport clinics	0 (0%)	0.00%, 0.02%	1,642 (2.4%)	2.3%, 2.6%	
Gouvernemental no Prenotif	0 (0%)	0.00%, 0.02%	58,064 (86%)	86%, 87%	
Gouvernmental with Prenotif	0 (0%)	0.00%, 0.02%	331 (0.5%)	0.44%, 0.55%	
Not Applicable	21,194 (100%)	100%, 100%	884 (1.3%)	1.2%, 1.4%	
Other	0 (0%)	0.00%, 0.02%	144 (0.2%)	0.18%, 0.25%	
PEC	0 (0%)	0.00%, 0.02%	5,763 (8.6%)	8.4%, 8.8%	
Private	0 (0%)	0.00%, 0.02%	457 (0.7%)	0.62%, 0.74%	
**PatientTriageArea**					**<0.001**
Adult_Assessement_ED	0 (0%)	0.00%, 0.02%	36,325 (54%)	54%, 54%	
ByPass_Crit_ED	0 (0%)	0.00%, 0.02%	3,703 (5.5%)	5.3%, 5.7%	
Dialysis	0 (0%)	0.00%, 0.02%	2 (<0.1%)	0.00%, 0.01%	
Low_Acuity_ED	0 (0%)	0.00%, 0.02%	16,713 (25%)	25%, 25%	
Not Applicable	21,194 (100%)	100%, 100%	1,490 (2.2%)	2.1%, 2.3%	
Ob_Gyn_ED	0 (0%)	0.00%, 0.02%	2,426 (3.6%)	3.5%, 3.7%	
Other	0 (0%)	0.00%, 0.02%	1,037 (1.5%)	1.5%, 1.6%	
Paed_ED	0 (0%)	0.00%, 0.02%	5,589 (8.3%)	8.1%, 8.5%	
**Asthma**	777 (3.7%)	3.4%, 3.9%	2,368 (3.5%)	3.4%, 3.7%	**0.3**
**CAD**	750 (3.5%)	3.3%, 3.8%	2,996 (4.5%)	4.3%, 4.6%	**<0.001**
**COPD**	64 (0.3%)	0.23%, 0.39%	261 (0.4%)	0.34%, 0.44%	**0.082**
**CVA**	140 (0.7%)	0.56%, 0.78%	911 (1.4%)	1.3%, 1.4%	**<0.001**
**Seizure**	170 (0.8%)	0.69%, 0.93%	1,096 (1.6%)	1.5%, 1.7%	**<0.001**
**DM**	2,678 (13%)	12%, 13%	9,093 (14%)	13%, 14%	**0.001**
**Hypertension**	2,768 (13%)	13%, 14%	9,953 (15%)	15%, 15%	**<0.001**
**None**	13,892 (66%)	65%, 66%	40,072 (60%)	59%, 60%	**<0.001**
**Others**	2,285 (11%)	10%, 11%	9,533 (14%)	14%, 14%	**<0.001**
**Surgeries**	360 (1.7%)	1.5%, 1.9%	1,797 (2.7%)	2.6%, 2.8%	**<0.001**
**Unknown**	810 (3.8%)	3.6%, 4.1%	4,489 (6.7%)	6.5%, 6.9%	**<0.001**
**Currently Pregnant**	306 (1.4%)	1.3%, 1.6%	2,884 (4.3%)	4.1%, 4.4%	**<0.001**
**Not Currently Pregnant**	7,725 (36%)	36%, 37%	19,387 (29%)	28%, 29%	

Wilcoxon rank-sum; Chi-squared test

2
