# Supplementary figures and images for: Predictive modelling of transport decisions and resources optimisation in pre-hospital setting using machine learning techniques
Source: PLoS One. 2024 May 3;19(5):e0301472. doi: 10.1371/journal.pone.0301472 (PMC11068197; doi:10.1371/journal.pone.0301472)

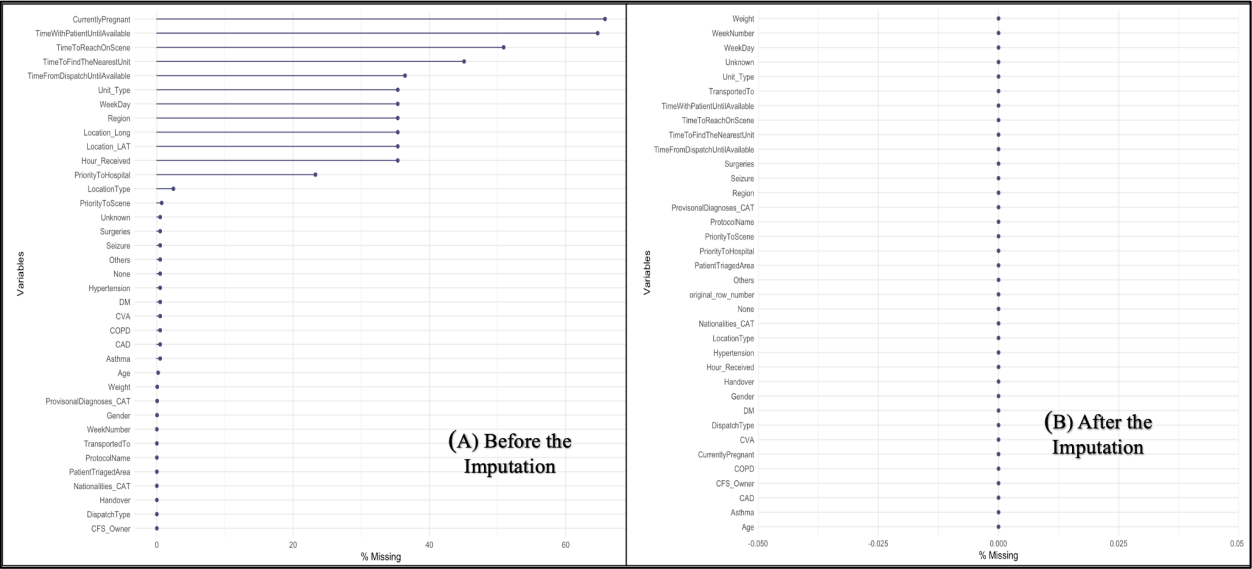

Supplement: S2 File — (PDF) [file pone.0301472.s002.pdf]

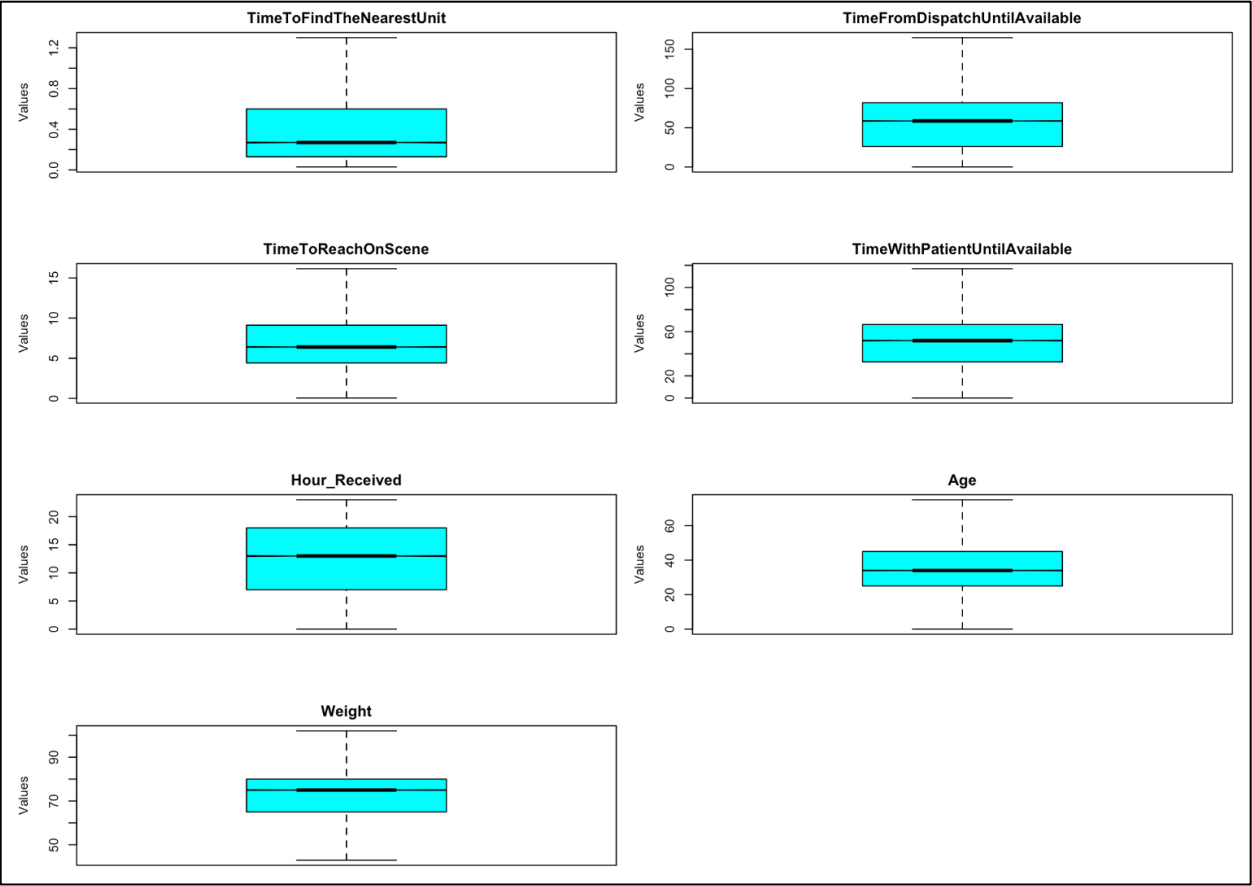

Supplement: S3 File — (PDF) [file pone.0301472.s003.pdf]
