# Supplementary material for: Predictive modelling of transport decisions and resources optimisation in pre-hospital setting using machine learning techniques
Source: PLoS One. 2024 May 3;19(5):e0301472. doi: 10.1371/journal.pone.0301472 (PMC11068197; doi:10.1371/journal.pone.0301472)
Supplement: S4 File — (PDF) [file pone.0301472.s004.pdf]

I. Random Forest Method

Variables' names	Not Transported	Transported	Mean Decrease Accuracy	Mean Decrease Gini
CFS_Owner	5.57	6.01	6.6	41.03
ProtocolName	7.62	4.48	8.13	198.9
DispatchType	6.2	2.71	4.98	88.3
PriorityToScene	5.51	2.76	4.32	14.68
PriorityToHospital	17.37	9.04	13.93	6756.69
Hour_Received	5.24	8.8	8.37	72.65
WeekDay	6.39	7.05	8.97	65.17
Region	5.97	7.16	7.5	19.85
LocationType	7.56	5.83	7.81	248.25
Gender	4.11	7.95	6.11	20.48
Age	6.4	7.3	9.32	87.52
Weight	4.76	7.5	7.17	84.6
Unit_Type	8.13	6.16	9.31	158.31
TransportedTo	12.16	9.41	11.93	6984.93
PatientTriageArea	10.37	10.27	11.02	7151.99
Asthma	2.7	0.58	2.53	6.5
CAD	3.32	2.44	3.9	6.19
COPD	0.16	2.02	1.72	3.88
CVA	1.69	2.6	2.48	5.04
Seizure	4.02	1.06	4.18	4.84
DM	4.02	4.65	5.16	9.73
Hypertension	2.09	4.18	3.96	9.7
None	1.17	6.09	3.63	24.95
Others	3.64	4.04	5.08	14.54
Surgeries	2.63	5.4	4.39	6.2
Unknown	3.55	7.07	6.17	24.82
CurrentlyPregnant	4.49	5.93	5.87	36.27
WeekNumber	6.75	4.47	6.69	96.99
TimeToFindTheNearestUnit	5.02	4.68	5.74	62.61
TimeToReachOnScene	3.54	4.018	4.69	81.59
TimeWithPatientUntilAvailable	7.5	6.16	8.61	442.32
TimeFromDispatchUntilAvailable	12.1	5.63	10.51	408.9
ProvisonalDiagnoses_CAT	17.53	8.9	16.87	344.18
Nationalities_CAT	6.11	8.99	9.22	150.06

II. Recursive Feature Elimination (RFE) method

Variable' numbers in the RFE	Root Mean Square Error	R^2	Mean Absolute Error	Root Mean Square Error Standard Deviation	R-squared Standard Deviation	Mean Absolute Error Standard Deviation
**1**	5.55×10^{-15}	1	4.78×10^{-15}	4.22×10^{-15}	2.47×10^{-15}	4.39×10^{-15}
**2**	2.30×10^{-02}	1	4.49×10^{-03}	4.51×10^{-03}	1.29×10^{-03}	7.56×10^{-04}
**3**	3.95×10^{-02}	0.99	9.05×10^{-03}	6.71×10^{-03}	3.03×10^{-03}	1.27×10^{-03}
**4**	3.89×10^{-02}	0.99	9.27×10^{-03}	5.87×10^{-03}	2.82×10^{-03}	1.25×10^{-03}
**5**	4.06×10^{-02}	0.99	1.07×10^{-02}	6.46×10^{-03}	3.21×10^{-03}	1.45×10^{-03}
**6**	1.16×10^{-02}	0.99	2.26×10^{-03}	1.77×10^{-03}	2.16×10^{-04}	3.93×10^{-04}
**7**	1.36×10^{-02}	1	2.81×10^{-03}	1.99×10^{-03}	3.18×10^{-04}	4.56×10^{-04}
**8**	1.57×10^{-02}	1	3.42×10^{-03}	3.27×10^{-03}	5.98×10^{-04}	6.53×10^{-04}

**9**	8.03×10^{-03}	1	1.48×10^{-03}	2.13×10^{-03}	1.83×10^{-04}	2.78×10^{-04}
**10**	9.91×10^{-03}	1	2.05×10^{-03}	1.58×10^{-03}	1.66×10^{-04}	3.41×10^{-04}
**11**	1.13×10^{-02}	1	2.46×10^{-03}	1.78×10^{-03}	2.23×10^{-04}	2.54×10^{-04}
**12**	7.42×10^{-03}	1	1.52×10^{-03}	2.12×10^{-03}	1.69×10^{-04}	3.51×10^{-04}
**13**	8.55×10^{-03}	1	1.83×10^{-03}	1.82×10^{-03}	1.62×10^{-04}	3.86×10^{-04}
**14**	9.36×10^{-03}	1	2.13×10^{-03}	1.82×10^{-03}	1.82×10^{-04}	3.40×10^{-04}
**15**	7.54×10^{-03}	1	1.57×10^{-03}	1.74×10^{-03}	1.43×10^{-04}	3.21×10^{-04}
**16**	8.40×10^{-03}	1	1.82×10^{-03}	2.03×10^{-03}	1.83×10^{-04}	4.33×10^{-04}
**17**	9.85×10^{-03}	1	2.22×10^{-03}	1.84×10^{-03}	2.02×10^{-04}	3.70×10^{-04}
**18**	7.21×10^{-03}	1	1.54×10^{-03}	1.89×10^{-03}	1.50×10^{-04}	3.66×10^{-04}
**19**	8.14×10^{-03}	1	1.79×10^{-03}	1.72×10^{-03}	1.62×10^{-04}	3.34×10^{-04}
**20**	9.71×10^{-03}	1	2.20×10^{-03}	2.14×10^{-03}	2.31×10^{-04}	4.48×10^{-04}
**21**	7.34×10^{-03}	1	1.60×10^{-03}	1.47×10^{-03}	1.13×10^{-04}	2.78×10^{-04}
**22**	8.64×10^{-03}	1	1.96×10^{-03}	1.68×10^{-03}	1.45×10^{-04}	3.28×10^{-04}
**23**	9.40×10^{-03}	1	2.08×10^{-03}	2.14×10^{-03}	2.35×10^{-04}	4.44×10^{-04}
**24**	7.39×10^{-03}	1	1.64×10^{-03}	1.97×10^{-03}	1.67×10^{-04}	3.43×10^{-04}
**25**	7.86×10^{-03}	1	1.80×10^{-03}	1.57×10^{-03}	1.33×10^{-04}	2.77×10^{-04}
**26**	9.01×10^{-03}	1	2.14×10^{-03}	1.81×10^{-03}	1.69×10^{-04}	4.05×10^{-04}
**27**	7.58×10^{-03}	1	1.65×10^{-03}	1.48×10^{-03}	1.17×10^{-04}	2.61×10^{-04}
**28**	8.05×10^{-03}	1	1.89×10^{-03}	1.72×10^{-03}	1.41×10^{-04}	4.47×10^{-04}
**29**	8.71×10^{-03}	1	2.10×10^{-03}	1.61×10^{-03}	1.45×10^{-04}	4.26×10^{-04}
**30**	7.29×10^{-03}	1	1.72×10^{-03}	1.87×10^{-03}	1.49×10^{-04}	3.88×10^{-04}
**35**	8.40×10^{-03}	1	2.05×10^{-03}	2.09×10^{-03}	1.94×10^{-04}	4.08×10^{-04}
**Recursive Feature Selection. Outer Resampling Method: Cross-Validated (10-Fold)**						
**The Top 4 Variables: Prioritytohospital, PATIENDTRIAGEAREA, PROVISONALDIAGNOSES_CAT, ProtocolName**						
