# Supplementary material for: Predictive modelling of transport decisions and resources optimisation in pre-hospital setting using machine learning techniques
Source: PLoS One. 2024 May 3;19(5):e0301472. doi: 10.1371/journal.pone.0301472 (PMC11068197; doi:10.1371/journal.pone.0301472)
Supplement: S5 File — (PDF) [file pone.0301472.s005.pdf]

I. XGBoost Algorithm predictions

Hour	Weekday	Yes	No	Weekday	Yes	No	Weekday	Yes	No	Weekday	Yes	No
**0**	Sunday	64	14	Monday	65	15	Tuesday	43	18	Wednesday	60	20
**1**	Sunday	33	6	Monday	51	21	Tuesday	45	21	Wednesday	59	19
**2**	Sunday	34	5	Monday	46	11	Tuesday	30	8	Wednesday	34	19
**3**	Sunday	25	3	Monday	24	7	Tuesday	28	6	Wednesday	41	9
**4**	Sunday	29	7	Monday	75	11	Tuesday	71	5	Wednesday	45	1
**5**	Sunday	33	4	Monday	32	8	Tuesday	42	5	Wednesday	39	10
**6**	Sunday	38	9	Monday	55	4	Tuesday	65	17	Wednesday	61	18
**7**	Sunday	48	2	Monday	52	10	Tuesday	47	10	Wednesday	69	17
**8**	Sunday	50	1	Monday	62	6	Tuesday	44	6	Wednesday	42	9
**9**	Sunday	48	6	Monday	82	7	Tuesday	52	9	Wednesday	72	6
**10**	Sunday	57	6	Monday	163	10	Tuesday	258	19	Wednesday	77	7
**11**	Sunday	155	22	Monday	341	30	Tuesday	150	12	Wednesday	446	61
**12**	Sunday	100	16	Monday	138	22	Tuesday	87	12	Wednesday	193	27
**13**	Sunday	72	10	Monday	109	16	Tuesday	83	6	Wednesday	136	31
**14**	Sunday	86	17	Monday	198	25	Tuesday	108	7	Wednesday	187	27
**15**	Sunday	64	6	Monday	59	10	Tuesday	64	11	Wednesday	54	10
**16**	Sunday	68	10	Monday	68	16	Tuesday	59	10	Wednesday	67	25
**17**	Sunday	65	8	Monday	113	20	Tuesday	59	10	Wednesday	85	15
**18**	Sunday	163	21	Monday	344	57	Tuesday	170	21	Wednesday	465	101
**19**	Sunday	77	10	Monday	69	25	Tuesday	64	10	Wednesday	85	19
**20**	Sunday	73	4	Monday	86	9	Tuesday	75	10	Wednesday	79	14
**21**	Sunday	57	10	Monday	114	27	Tuesday	49	18	Wednesday	79	25
**22**	Sunday	76	14	Monday	84	21	Tuesday	81	10	Wednesday	57	9
**23**	Sunday	54	14	Monday	76	15	Tuesday	38	13	Wednesday	61	17

Hour	Weekday	Yes	No	Weekday	Yes	No	Weekday	Yes	No
**0**	Thursday	41	11	Friday	47	24	Saturday	67	20
**1**	Thursday	34	13	Friday	60	14	Saturday	59	5
**2**	Thursday	19	10	Friday	24	16	Saturday	72	7
**3**	Thursday	30	9	Friday	20	7	Saturday	31	2
**4**	Thursday	30	0	Friday	31	3	Saturday	39	1
**5**	Thursday	47	4	Friday	81	6	Saturday	58	13
**6**	Thursday	44	12	Friday	28	7	Saturday	37	5
**7**	Thursday	51	7	Friday	35	9	Saturday	41	5
**8**	Thursday	61	7	Friday	33	3	Saturday	49	3
**9**	Thursday	49	7	Friday	39	2	Saturday	44	6
**10**	Thursday	61	10	Friday	56	1	Saturday	55	8
**11**	Thursday	227	37	Friday	33	3	Saturday	685	73
**12**	Thursday	153	11	Friday	65	6	Saturday	308	25
**13**	Thursday	111	20	Friday	50	3	Saturday	115	23
**14**	Thursday	166	34	Friday	69	9	Saturday	323	46
**15**	Thursday	67	20	Friday	78	13	Saturday	49	9
**16**	Thursday	86	19	Friday	59	10	Saturday	61	17
**17**	Thursday	77	11	Friday	55	8	Saturday	63	22
**18**	Thursday	367	42	Friday	62	12	Saturday	854	103
**19**	Thursday	79	9	Friday	59	14	Saturday	52	18
**20**	Thursday	75	20	Friday	58	21	Saturday	74	25
**21**	Thursday	86	31	Friday	63	12	Saturday	76	21
**22**	Thursday	88	15	Friday	102	12	Saturday	80	7
**23**	Thursday	83	14	Friday	45	5	Saturday	49	15

II. Random Forest predictions

**Hour**	**Weekday**	**Yes**	**No**	**Weekday**	**Yes**	**No**	**Weekday**	**Yes**	**No**	**Weekday**	**Yes**	**No**
**0**	Sunday	64	14	Monday	70	0	Tuesday	46	15	Wednesday	63	17
**1**	Sunday	36	3	Monday	55	1	Tuesday	54	12	Wednesday	58	20
**2**	Sunday	36	3	Monday	45	2	Tuesday	31	7	Wednesday	36	17
**3**	Sunday	26	2	Monday	29	3	Tuesday	29	5	Wednesday	44	6
**4**	Sunday	30	6	Monday	75	4	Tuesday	74	2	Wednesday	43	3
**5**	Sunday	33	4	Monday	34	5	Tuesday	44	3	Wednesday	43	6
**6**	Sunday	39	8	Monday	56	6	Tuesday	69	13	Wednesday	61	18
**7**	Sunday	47	3	Monday	56	7	Tuesday	48	9	Wednesday	70	16
**8**	Sunday	51	0	Monday	65	8	Tuesday	44	6	Wednesday	45	6
**9**	Sunday	50	4	Monday	83	9	Tuesday	55	6	Wednesday	73	5
**10**	Sunday	60	3	Monday	165	10	Tuesday	263	14	Wednesday	77	7
**11**	Sunday	163	14	Monday	355	11	Tuesday	155	7	Wednesday	465	42
**12**	Sunday	106	10	Monday	152	12	Tuesday	90	9	Wednesday	195	25
**13**	Sunday	79	3	Monday	114	13	Tuesday	86	3	Wednesday	151	16
**14**	Sunday	91	12	Monday	207	14	Tuesday	111	4	Wednesday	193	21
**15**	Sunday	65	5	Monday	62	15	Tuesday	66	9	Wednesday	56	8
**16**	Sunday	72	6	Monday	77	16	Tuesday	61	8	Wednesday	70	22
**17**	Sunday	67	6	Monday	115	17	Tuesday	62	7	Wednesday	91	9
**18**	Sunday	171	13	Monday	365	18	Tuesday	176	15	Wednesday	497	69
**19**	Sunday	80	7	Monday	74	19	Tuesday	66	8	Wednesday	89	15
**20**	Sunday	72	5	Monday	87	20	Tuesday	77	8	Wednesday	83	10
**21**	Sunday	60	7	Monday	119	21	Tuesday	54	13	Wednesday	82	22
**22**	Sunday	81	9	Monday	89	22	Tuesday	85	6	Wednesday	56	10
**23**	Sunday	56	12	Monday	78	23	Tuesday	45	6	Wednesday	63	15

**Hour**	**Weekday**	**Yes**	**No**	**Weekday**	**Yes**	**No**	**Weekday**	**Yes**	**No**
**0**	Thursday	44	8	Friday	53	18	Saturday	70	17
**1**	Thursday	37	10	Friday	59	15	Saturday	61	3
**2**	Thursday	22	7	Friday	33	7	Saturday	75	4
**3**	Thursday	33	6	Friday	23	4	Saturday	33	0
**4**	Thursday	30	0	Friday	30	4	Saturday	39	1
**5**	Thursday	49	2	Friday	81	6	Saturday	58	13
**6**	Thursday	46	10	Friday	30	5	Saturday	37	5
**7**	Thursday	51	7	Friday	33	11	Saturday	41	5
**8**	Thursday	63	5	Friday	35	1	Saturday	50	2
**9**	Thursday	54	2	Friday	38	3	Saturday	45	5
**10**	Thursday	64	7	Friday	56	1	Saturday	55	8
**11**	Thursday	244	20	Friday	33	3	Saturday	704	54
**12**	Thursday	156	8	Friday	67	4	Saturday	311	22
**13**	Thursday	114	17	Friday	50	3	Saturday	122	16
**14**	Thursday	173	27	Friday	75	3	Saturday	337	32
**15**	Thursday	74	13	Friday	84	7	Saturday	49	9
**16**	Thursday	92	13	Friday	63	6	Saturday	69	9
**17**	Thursday	81	7	Friday	58	5	Saturday	64	21
**18**	Thursday	377	32	Friday	67	7	Saturday	893	64
**19**	Thursday	81	7	Friday	58	15	Saturday	55	15
**20**	Thursday	81	14	Friday	67	12	Saturday	79	20
**21**	Thursday	93	24	Friday	65	10	Saturday	80	17
**22**	Thursday	90	13	Friday	105	9	Saturday	77	10
**23**	Friday	88	9		47	3	Saturday	54	10

III. AdaBoost Algorithm predictions

**Hour**	**WeekDay**	**Yes**	**No**	**WeekDay**	**Yes**	**No**	**WeekDay**	**Yes**	**No**	**WeekDay**	**Yes**	**No**
**0**	Friday	68	3	Sunday	70	8	Wednesday	72	8	Tuesday	58	3

**1**	Friday	70	4	Sunday	37	2	Wednesday	73	5	Tuesday	60	6
**2**	Friday	39	1	Sunday	39	0	Wednesday	51	2	Tuesday	37	1
**3**	Friday	25	2	Sunday	25	3	Wednesday	49	1	Tuesday	28	6
**4**	Friday	34	0	Sunday	32	4	Wednesday	43	3	Tuesday	75	1
**5**	Friday	84	3	Sunday	35	2	Wednesday	47	2	Tuesday	45	2
**6**	Friday	29	6	Sunday	40	7	Wednesday	78	1	Tuesday	79	3
**7**	Friday	39	5	Sunday	43	7	Wednesday	79	7	Tuesday	50	7
**8**	Friday	36	0	Sunday	51	0	Wednesday	51	0	Tuesday	48	2
**9**	Friday	41	0	Sunday	54	0	Wednesday	77	1	Tuesday	57	4
**10**	Friday	55	2	Sunday	62	1	Wednesday	83	1	Tuesday	277	0
**11**	Friday	36	0	Sunday	173	4	Wednesday	501	6	Tuesday	159	3
**12**	Friday	71	0	Sunday	113	3	Wednesday	215	5	Tuesday	98	1
**13**	Friday	53	0	Sunday	80	2	Wednesday	164	3	Tuesday	88	1
**14**	Friday	76	2	Sunday	97	6	Wednesday	210	4	Tuesday	112	3
**15**	Friday	90	1	Sunday	68	2	Wednesday	63	1	Tuesday	75	0
**16**	Friday	68	1	Sunday	73	5	Wednesday	88	4	Tuesday	66	3
**17**	Friday	62	1	Sunday	69	4	Wednesday	94	6	Tuesday	66	3
**18**	Friday	72	2	Sunday	180	4	Wednesday	553	13	Tuesday	188	3
**19**	Friday	70	3	Sunday	85	2	Wednesday	101	3	Tuesday	72	2
**20**	Friday	79	0	Sunday	76	1	Wednesday	92	1	Tuesday	83	2
**21**	Friday	73	2	Sunday	65	2	Wednesday	99	5	Tuesday	65	2
**22**	Friday	110	4	Sunday	86	4	Wednesday	66	0	Tuesday	88	3
**23**	Friday	49	1	Sunday	64	4	Wednesday	74	4	Tuesday	51	0

**Hour**	**Weekday**	**Yes**	**No**	**Weekday**	**Yes**	**No**	**Weekday**	**Yes**	**No**
**0**	Monday	74	6	Thursday	47	5	Saturday	81	6
**1**	Monday	67	5	Thursday	44	3	Saturday	64	0
**2**	Monday	51	6	Thursday	25	4	Saturday	77	0
**3**	Monday	30	1	Thursday	36	3	Saturday	32	0
**4**	Monday	84	2	Thursday	30	0	Saturday	40	0
**5**	Monday	37	3	Thursday	50	1	Saturday	67	0
**6**	Monday	56	3	Thursday	47	9	Saturday	38	0
**7**	Monday	58	4	Thursday	55	3	Saturday	44	0
**8**	Monday	66	2	Thursday	64	4	Saturday	50	0
**9**	Monday	88	1	Thursday	54	2	Saturday	48	0
**10**	Monday	172	1	Thursday	68	3	Saturday	62	0
**11**	Monday	364	7	Thursday	261	3	Saturday	748	0
**12**	Monday	155	5	Thursday	160	4	Saturday	330	3
**13**	Monday	123	2	Thursday	130	1	Saturday	135	3
**14**	Monday	220	3	Thursday	198	2	Saturday	364	5
**15**	Monday	66	3	Thursday	84	3	Saturday	58	0
**16**	Monday	81	3	Thursday	104	1	Saturday	77	1
**17**	Monday	127	6	Thursday	86	2	Saturday	82	3
**18**	Monday	391	10	Thursday	398	11	Saturday	943	14
**19**	Monday	91	3	Thursday	85	3	Saturday	66	4
**20**	Monday	93	2	Thursday	90	5	Saturday	94	5
**21**	Monday	138	3	Thursday	113	4	Saturday	96	1
**22**	Monday	103	2	Thursday	103	0	Saturday	86	1
**23**	Monday	87	4	Thursday	95	2	Saturday	64	0

IV. SVM Algorithm predictions

**Hour**	**Weekday**	**Yes**	**No**	**Weekday**	**Yes**	**No**	**Weekday**	**Yes**	**No**	**Weekday**	**Yes**	**No**
**0**	Friday	68	3	Friday	81	6	Thursday	47	5	Wednesday	72	8
**1**	Friday	70	4	Saturday	64	0	Thursday	44	3	Wednesday	73	5
**2**	Friday	39	1	Saturday	77	2	Thursday	25	4	Wednesday	51	2

**3**	Friday	25	2	Saturday	32	1	Thursday	36	3	Wednesday	49	1
**4**	Friday	34	0	Saturday	40	0	Thursday	30	0	Wednesday	43	3
**5**	Friday	84	3	Saturday	67	4	Thursday	50	1	Wednesday	47	2
**6**	Friday	29	6	Saturday	38	4	Thursday	47	9	Wednesday	78	1
**7**	Friday	39	5	Saturday	44	2	Thursday	55	3	Wednesday	79	7
**8**	Friday	36	0	Saturday	50	2	Thursday	64	4	Wednesday	51	0
**9**	Friday	41	0	Saturday	48	2	Thursday	54	2	Wednesday	77	1
**10**	Friday	55	2	Saturday	62	1	Thursday	68	3	Wednesday	83	1
**11**	Friday	36	0	Saturday	748	10	Thursday	261	3	Wednesday	501	6
**12**	Friday	71	0	Saturday	330	3	Thursday	160	4	Wednesday	215	5
**13**	Friday	53	0	Saturday	135	3	Thursday	130	1	Wednesday	164	3
**14**	Friday	76	2	Saturday	364	5	Thursday	198	2	Wednesday	210	4
**15**	Friday	90	1	Saturday	58	0	Thursday	84	3	Wednesday	63	1
**16**	Friday	68	1	Saturday	77	1	Thursday	104	1	Wednesday	88	4
**17**	Friday	62	1	Saturday	82	3	Thursday	86	2	Wednesday	94	6
**18**	Friday	72	2	Saturday	943	14	Thursday	398	11	Wednesday	553	13
**19**	Friday	70	3	Saturday	66	4	Thursday	85	3	Wednesday	101	3
**20**	Friday	79	0	Saturday	94	5	Thursday	90	5	Wednesday	92	1
**21**	Friday	73	2	Saturday	96	1	Thursday	113	4	Wednesday	99	5
**22**	Friday	110	4	Saturday	86	1	Thursday	103	0	Wednesday	66	0
**23**	Friday	49	1	Saturday	64	0	Thursday	95	2	Wednesday	74	4

**Hour**	**Weekday**	**Yes**	**No**	**Weekday**	**Yes**	**No**	**Weekday**	**Yes**	**No**
**0**	Monday	74	6	Sunday	70	8	Tuesday	58	3
**1**	Monday	67	5	Sunday	37	2	Tuesday	60	6
**2**	Monday	51	6	Sunday	39	0	Tuesday	37	1
**3**	Monday	30	1	Sunday	25	3	Tuesday	28	6
**4**	Monday	84	2	Sunday	32	4	Tuesday	75	1
**5**	Monday	37	3	Sunday	35	2	Tuesday	45	2
**6**	Monday	56	3	Sunday	40	7	Tuesday	79	3
**7**	Monday	58	4	Sunday	43	7	Tuesday	50	7
**8**	Monday	66	2	Sunday	51	0	Tuesday	48	2
**9**	Monday	88	1	Sunday	54	0	Tuesday	57	4
**10**	Monday	172	1	Sunday	62	1	Tuesday	277	0
**11**	Monday	364	7	Sunday	173	4	Tuesday	159	3
**12**	Monday	155	5	Sunday	113	3	Tuesday	98	1
**13**	Monday	123	2	Sunday	80	2	Tuesday	88	1
**14**	Monday	220	3	Sunday	97	6	Tuesday	112	3
**15**	Monday	66	3	Sunday	68	2	Tuesday	75	0
**16**	Monday	81	3	Sunday	73	5	Tuesday	66	3
**17**	Monday	127	6	Sunday	69	4	Tuesday	66	3
**18**	Monday	391	10	Sunday	180	4	Tuesday	188	3
**19**	Monday	91	3	Sunday	85	2	Tuesday	72	2
**20**	Monday	93	2	Sunday	76	1	Tuesday	83	2
**21**	Monday	138	3	Sunday	65	2	Tuesday	65	2
**22**	Monday	103	2	Sunday	86	4	Tuesday	88	3
**23**	Monday	87	4	Sunday	64	4	Tuesday	51	0
