# Supplementary material for: Predictive modelling of transport decisions and resources optimisation in pre-hospital setting using machine learning techniques
Source: PLoS One. 2024 May 3;19(5):e0301472. doi: 10.1371/journal.pone.0301472 (PMC11068197; doi:10.1371/journal.pone.0301472)
Supplement: S6 File — (PDF) [file pone.0301472.s006.pdf]

```r
# Importing the required packages
library(caret)
library(doParallel)
library(e1071)
library(xgboost)
library(MLmetrics)
library(rpart)
library(ada)
library(fastAdaboost)
library(caTools)
library(kableExtra)
library(dplyr)
library(ggplot2)
# RawData selected variables for the ML
colnames(RawData)
RawData <- subset(RawData, select = c("Handover", "Unit_Type", "Region", "ProtocolName", "WeekNumber", "Hour_Received", "LocationType", "WeekDay", "Age", "Gender", "Nationalities_CAT", "ProvisonalDiagnoses_CAT", "Hypertension", "DM"))
colnames(RawData)
# Setting the seed for reproducibility
set.seed(123)
# Creating training and test indices
trainIndex <- createDataPartition(RawData$Handover, p = .8, list = FALSE, times = 1)
# Splitting the data into training and testing sets
trainData <- RawData[trainIndex,]
testData <- RawData[-trainIndex,]
# Setting levels for ProtocolName in both train and test data
all_levels <- unique(c(levels(trainData$ProtocolName), levels(testData$ProtocolName)))
trainData$ProtocolName <- factor(trainData$ProtocolName, levels = all_levels)
testData$ProtocolName <- factor(testData$ProtocolName, levels = all_levels)
testData$ProtocolName[is.na(testData$ProtocolName)] <- "DefaultValue"
# Converting categorical variables to factors
trainData$Nationalities_CAT <- as.factor(trainData$Nationalities_CAT)
trainData$Gender <- as.factor(trainData$Gender)
testData$Nationalities_CAT <- as.factor(testData$Nationalities_CAT)
testData$Gender <- as.factor(testData$Gender)
# Converting numeric variables
trainData$Hypertension <- as.numeric(trainData$Hypertension)
trainData$DM <- as.numeric(trainData$DM)
testData$Hypertension <- as.numeric(testData$Hypertension)
testData$DM <- as.numeric(testData$DM)
# Setting levels for WeekNumber
trainData$WeekNumber <- factor(trainData$WeekNumber)
testData$WeekNumber <- factor(testData$WeekNumber, levels = levels(trainData$WeekNumber))
levels(trainData$WeekNumber) <- c(levels(trainData$WeekNumber), "OtherWeek")
levels(testData$WeekNumber) <- levels(trainData$WeekNumber)
new_levels <- setdiff(levels(testData$WeekNumber), levels(trainData$WeekNumber))
if(length(new_levels) > 0) {
  testData$WeekNumber[testData$WeekNumber %in% new_levels] <- "OtherWeek"
}
testData$WeekNumber <- as.factor(testData$WeekNumber)
trainData$WeekNumber <- as.factor(trainData$WeekNumber)
# Removing rows containing missing values
```

```
trainData <- na.omit(trainData)
testData <- na.omit(testData)
# Parallel computing
registerDoParallel(cores = detectCores())
# Cross-validation and hyperparameter tuning
control <- trainControl(method = "cv", number = 10, allowParallel = TRUE)
# Set tuning parameters
tunegrid <- expand.grid(mtry = c(1:10))
xgbGrid <- expand.grid(nrounds = 100, eta = c(0.01, 0.1, 0.3), max_depth = c(2, 6, 10), gamma = 0, colsample_bytree = c(0.6, 0.8, 1), min_child_weight = 1, subsample = 1)
# Training the Models
set.seed(123)
formula <- as.formula(Handover ~ .)
model_rf <- train(formula, data = trainData, method = "rf", trControl = control, tuneGrid = tunegrid)
model_svm <- svm(Handover ~ ., data = trainData, kernel = "radial")
model_xgb <- train(formula, data = trainData, method = "xgbTree", trControl = control, tuneGrid = xgbGrid)
trainData <- as.data.frame(trainData)
model_adb <- ada(Handover ~ ., data = trainData, iter = 100)
print("--------------------------------------------")
# Creating a list of the models
models_list <- list(Random_Forest = model_rf,
                    Support_Vector_Machine = model_svm,
                    XGBoost = model_xgb,
                    AdaBoost = model_adb)
# Creating models colour map
color_map <- c("Random_Forest" = "red",
               "Support_Vector_Machine" = "blue",
               "XGBoost" = "green",
               "AdaBoost" = "purple")

# Initialising an empty list to store evaluation results
evaluation_results <- list()
# Looping over models to evaluate them
for(model_name in names(models_list)) {
  model <- models_list[[model_name]]
  # Predictions
  preds <- predict(model, newdata = testData)
  # Models that support probability estimates
  if(model_name %in% c('Random_Forest', 'XGBoost')) {
    pred_probs <- as.numeric(predict(model, newdata = testData, type='prob')[,2])
  } else {
    pred_probs <- NULL
  }
  # Confusion matrix
  cm <- confusionMatrix(preds, testData$Handover)
  # Other various Metrics
  accuracy <- cm$overall['Accuracy']
  sensitivity <- cm$byClass['Sensitivity']
  specificity <- cm$byClass['Specificity']
  recall <- sensitivity
  f1 <- 2 * (sensitivity * specificity) / (sensitivity + specificity)
  mcc <- mcc(preds, testData$Handover)
```

```
  # ROC and AUC
  if (!is.null(pred_probs)) {
    roc_obj <- pROC::roc(testData$Handover, pred_probs)
    auc <- as.numeric(pROC::auc(roc_obj))
  } else {
    roc_obj <- NULL
    auc <- NULL
  }

  # Storing the results un the empty list
  evaluation_results[[model_name]] <- list(
    Confusion_Matrix = cm,
    Accuracy = accuracy,
    Sensitivity = sensitivity,
    Specificity = specificity,
    Recall = recall,
    F1 = f1,
    MCC = mcc,
    AUC = auc
  )
  if (!is.null(roc_obj)) {
    pROC::plot.roc(roc_obj, main=paste("ROC for ", model_name), col=color_map[model_name])
    text(0.7, 0.2, paste0("AUC = ", round(auc, 2)), adj = 1)
  }
}
print(evaluation_results)
print("-------------------------------------------")
# Creating an empty data frame to hold the metrics
result_df <- data.frame()
# The list of evaluation results
for(model_name in names(evaluation_results)) {
  temp_df <- data.frame(
    Model = model_name,
    Accuracy = evaluation_results[[model_name]]$Accuracy,
    Sensitivity = evaluation_results[[model_name]]$Sensitivity,
    Specificity = evaluation_results[[model_name]]$Specificity,
    Recall = evaluation_results[[model_name]]$Recall,
    F1 = evaluation_results[[model_name]]$F1,
    MCC = evaluation_results[[model_name]]$MCC,
    AUC = ifelse(is.null(evaluation_results[[model_name]]$AUC), NA,
evaluation_results[[model_name]]$AUC)
  )
  result_df <- rbind(result_df, temp_df)
}
print(result_df)


print("---------------------------------------------------------------------------------------------------------")
#Feature Importance
# Defining a function to compute permutation feature importance
perm_feature_importance <- function(model, data, outcome) {
  feature_importance <- data.frame(Predictor = character(), Importance = numeric())
  baseline_accuracy <- sum(predict(model, newdata = data) == data[[outcome]]) / nrow(data)
```

```
  for (predictor in setdiff(names(data), outcome)) {
    perm_data <- data
    perm_data[[predictor]] <- sample(data[[predictor]])
    perm_accuracy <- sum(predict(model, newdata = perm_data) == data[[outcome]]) / nrow(data)
    importance <- baseline_accuracy - perm_accuracy
    feature_importance <- rbind(feature_importance, data.frame(Predictor = predictor, Importance = importance))
  }
  return(feature_importance)
}
# Calculating
models_list <- list(Random_Forest = model_rf, Support_Vector_Machine = model_svm, XGBoost =
model_xgb, AdaBoost=model_adb)
feature_importances_list <- lapply(models_list, perm_feature_importance, data = testData, outcome =
"Handover")
print(feature_importances_list)
# importing the data of each model to a dataframe
feature_importances_df <- data.frame(Predictor = character(), Importance = numeric(), Model = character())
for(i in seq_along(feature_importances_list)) {
  feature_importance <- feature_importances_list[[i]]
  model_name <- names(feature_importances_list)[i]
  df <- data.frame(feature_importance, Model = model_name, stringsAsFactors = FALSE)
  feature_importances_df <- rbind(feature_importances_df, df)
}
feature_importances_gt <- gt(feature_importances_df) %>%
  fmt_number(
    columns = vars(Importance),
    decimals = 2
  )
print(feature_importances_gt)
html_output <- as_raw_html(feature_importances_gt)
write(html_output, "feature_importances.html")
#Plotting the feature importance
feature_importances_df <- feature_importances_df[order(feature_importances_df$Importance, decreasing =
TRUE),]
theme_set(theme_gray(base_size = 15))
ggplot(feature_importances_df, aes(x = Predictor, y = Importance, fill = Model, label = round(Importance, 2)))
+
  geom_bar(stat = "identity", position = position_dodge()) +
  geom_text(size = 4, position = position_dodge(0.9), hjust = -0.1) +
  theme(axis.text.x = element_text(angle = 0, vjust = 0.5, hjust=1),
        axis.text.y = element_text(size = 12),
        title = element_text(size = 16),
        axis.title = element_text(size = 12)) +
  labs(x = "Features", y = "Importance", title = "Feature importance by Model", fill = "Model") +
  scale_fill_brewer(palette = "Set1") + coord_flip()
print("--------------------------------------------------------------------------------------------------------")
#Predicting (Using Quality Control Tools)
testData$prediction_rf <- predict(model_rf, newdata=testData[, -which(names(testData) == "Handover")])
testData$prediction_svm <- predict(model_svm, newdata=testData[, -which(names(testData) == "Handover")])
testData$prediction_xgb <- predict(model_xgb, newdata=testData[, -which(names(testData) == "Handover")])
testData$prediction_adb <- predict(model_adb, newdata=testData[, -which(names(testData) == "Handover")])
colnames(testData)
p_chart <- function(data, prediction_col, model_name) {
```

```
  agg_data <- data %>%
    group_by(Hour_Received) %>%
    summarize(not_transported = sum(ifelse(Handover == 0 & !!sym(prediction_col) == 0, 1, 0)),
              total = n()) %>%
    mutate(proportion = not_transported / total)

  qcc(agg_data$proportion, sizes = agg_data$total, type = "p",
      xlab = "Hour_Received", ylab = "Proportion of Not Transported",
      main = paste("Control p-chart for", model_name))
}
c_chart <- function(data, prediction_col, model_name) {
  agg_data <- data %>%
    group_by(Hour_Received) %>%
    summarize(not_transported = sum(ifelse(Handover == 0 & !!sym(prediction_col) == 0, 1, 0)),
              total = n())

  qcc(agg_data$not_transported, type = "c",
      xlab = "Hour_Received", ylab = "Count of Not Transported",
      main = paste("Control c-chart for", model_name))
}
plot1 <- p_chart(testData, "prediction_xgb", "XGB")
plot2 <- c_chart(testData, "prediction_xgb", "XGB")
plot3 <- p_chart(testData, "prediction_rf", "RF")
plot4 <- c_chart(testData, "prediction_rf", "RF")
plot5 <- p_chart(testData, "prediction_svm", "SVM")
plot6 <- c_chart(testData, "prediction_svm", "SVM")
plot1 <- p_chart(testData, "prediction_adb", "AdaBoost")
plot7 <- c_chart(testData, "prediction_adb", "AdaBoost")
print("---------------------------------------------------------------------------------------------------")
# Creating a function to generate summary for each hour, weekday, and each model
summarize_predictions_by_hour_and_weekday <- function(data, prediction_col, model_name) {
  summary_by_hour_weekday <- data %>%
    group_by(WeekDay, Hour_Received) %>%
    summarise(Handed_Over = sum(get(prediction_col) == 1),
              Not_Handed_Over = sum(get(prediction_col) == 0)) %>%
    mutate(Model = model_name)

  return(summary_by_hour_weekday)
}
hourly_weekday_summary <- data.frame()
prediction_cols <- c("prediction_rf", "prediction_svm", "prediction_xgb", "prediction_adb")
model_names <- c("Random Forest", "SVM", "XGBoost", "AdaBoost")
for(i in 1:length(prediction_cols)) {
  temp_summary <- summarize_predictions_by_hour_and_weekday(testData, prediction_cols[i],
model_names[i])
  hourly_weekday_summary <- rbind(hourly_weekday_summary, temp_summary)
}
print(hourly_weekday_summary)
ggplot(hourly_weekday_summary, aes(x = Hour_Received, y = Handed_Over, fill = Model)) +
  geom_bar(stat = "identity", position = "dodge") +
  facet_wrap(~ WeekDay) +
  ylab("Number of Patients Handed Over") +
  xlab("Hour Received") +
```

```
  ggtitle("Hourly and Weekday Prediction of Number of Patients to be Handed Over") +
  theme_minimal()
# Generating tables for each model
random_forest_summary <- hourly_weekday_summary %>%
  filter(Model == "Random Forest")
print("Random Forest Summary")
print(random_forest_summary)
svm_summary <- hourly_weekday_summary %>%
  filter(Model == "SVM")
print("SVM Summary")
print(svm_summary)
xgboost_summary <- hourly_weekday_summary %>%
  filter(Model == "XGBoost")
print("XGBoost Summary")
print(xgboost_summary)
adaboost_summary <- hourly_weekday_summary %>%
  filter(Model == "AdaBoost")
print("AdaBoost Summary")
print(adaboost_summary)
write.csv(random_forest_summary, "Random_Forest_Summary.csv")
write.csv(svm_summary, "SVM_Summary.csv")
write.csv(xgboost_summary, "XGBoost_Summary.csv")
write.csv(adaboost_summary, "AdaBoost_Summary.csv")
```
